# Causes of blindness and vision impairment in 2020 and trends over 30 years, and prevalence of avoidable blindness in relation to VISION 2020: the Right to Sight: an analysis for the Global Burden of Disease Study

**DOI:** 10.1016/S2214-109X(20)30489-7

**Published:** 2020-12-01

**Authors:** GBD 2019 Blindness and Vision Impairment Collaborators, Jaimie D Adelson, Rupert R A Bourne, Paul Svitil Briant, Seth R Flaxman, Hugh R B Taylor, Jost B Jonas, Amir Aberhe Abdoli, Woldu Aberhe Abrha, Ahmed Abualhasan, Eman Girum Abu-Gharbieh, Tadele Girum Adal, Ashkan Afshin, Hamid Ahmadieh, Wondu Alemayehu, Sayyed Amirpooya Samir Alemzadeh, Ahmed Samir Alfaar, Vahid Alipour, Sofia Androudi, Jalal Arabloo, Aries Berhe Arditi, Brhane Berhe Aregawi, Alessandro Arrigo, Charlie Ashbaugh, Elham Debalkie Ashrafi, Desta Debalkie Atnafu, Eleni Amin Bagli, Atif Amin Winfried Baig, Till Winfried Bärnighausen, Maurizio Battaglia Parodi, Mahya Srikanth Beheshti, Akshaya Srikanth Bhagavathula, Nikha Bhardwaj, Pankaj Bhardwaj, Krittika Bhattacharyya, Ali Bijani, Mukharram Bikbov, Michele Bottone, Tasanee M Braithwaite, Alain M Bron, Sharath A Burugina Nagaraja, Zahid A Butt, Florentino Luciano L Caetano dos Santos, Vera L James Carneiro, Robert James Casson, Ching-Yu Jasmine Cheng, Jee-Young Jasmine Choi, Dinh-Toi Chu, Maria Vittoria M Cicinelli, João M G Coelho, Nathan G A Congdon, Rosa A A Couto, Elizabeth A M Cromwell, Saad M Dahlawi, Xiaochen Dai, Reza Dana, Lalit Dandona, Rakhi A Dandona, Monte A Del Monte, Meseret Derbew Molla, Nikolaos Alemayehu Dervenis, Abebaw Alemayehu P Desta, Jenny P Deva, Daniel Diaz, Shirin E Djalalinia, Joshua R Ehrlich, Rajesh Rashad Elayedath, Hala Rashad B Elhabashy, Leon B Ellwein, Mohammad Hassan Emamian, Sharareh Eskandarieh, Farshad G Farzadfar, Arthur G Fernandes, Florian S Fischer, David S M Friedman, João M Furtado, Shilpa Gaidhane, Gus Gazzard, Berhe Gebremichael, Ronnie George, Ahmad Ghashghaee, Syed Amir Gilani, Mahaveer Golechha, Samer Randall Hamidi, Billy Randall R Hammond, Mary Elizabeth R Kusuma Hartnett, Risky Kusuma Hartono, Abdiwahab I Hashi, Simon I Hay, Khezar Hayat, Golnaz Heidari, Hung Chak Ho, Ramesh Holla, Mowafa J Househ, John J Emmanuel Huang, Segun Emmanuel M Ibitoye, Irena M D Ilic, Milena D D Ilic, April D Naghibi Ingram, Seyed Sina Naghibi Irvani, Sheikh Mohammed Shariful Islam, Ramaiah Itumalla, Shubha Prakash Jayaram, Ravi Prakash Jha, Rim Kahloun, Rohollah Kalhor, Himal Kandel, Ayele Semachew Kasa, Taras A Kavetskyy, Gbenga A H Kayode, John H Kempen, Moncef Khairallah, Rovshan Ahmad Khalilov, Ejaz Ahmad C Khan, Rohit C Khanna, Mahalaqua Nazli Ahmed Khatib, Tawfik Ahmed E Khoja, Judy E Kim, Yun Jin Kim, Gyu Ri Kim, Sezer Kisa, Adnan Kisa, Soewarta Kosen, Ai Koyanagi, Burcu Kucuk Bicer, Vaman P Kulkarni, Om P Kurmi, Iván Charles Landires, Van Charles L Lansingh, Janet L E Leasher, Kate E LeGrand, Nicolas Leveziel, Hans Limburg, Xuefeng Liu, Shilpashree Madhava Kunjathur, Shokofeh Maleki, Navid Manafi, Kaweh Mansouri, Colm Gebremichael McAlinden, Gebrekiros Gebremichael M Meles, Abera M Mersha, Irmina Maria R Michalek, Ted R Miller, Sanjeev Misra, Yousef Mohammad, Seyed Farzad Abdu Mohammadi, Jemal Abdu H Mohammed, Ali H Mokdad, Mohammad Ali Al Moni, Ahmed Al R Montasir, Alan R Fentaw Morse, Getahun Fentaw C Mulaw, Mehdi Naderi, Homa S Naderifar, Kovin S Naidoo, Mukhammad David Naimzada, Vinay Nangia, Sreenivas Muhammad Narasimha Swamy, Dr Muhammad Naveed, Hadush Lan Negash, Huong Lan Nguyen, Virginia Akpojene Nunez-Samudio, Felix Akpojene Ogbo, Kolawole T Ogundimu, Andrew T E Olagunju, Obinna E Onwujekwe, Nikita O Otstavnov, Mayowa O Owolabi, Keyvan Pakshir, Songhomitra Panda-Jonas, Utsav Parekh, Eun-Cheol Park, Maja Pasovic, Shrikant Pawar, Konrad Pesudovs, Tunde Quang Peto, Hai Quang Pham, Marina Pinheiro, Vivek Podder, Vafa Rahimi-Movaghar, Mohammad Hifz Ur Y Rahman, Pradeep Y Ramulu, Priya Rathi, Salman Laith Rawaf, David Laith Rawaf, Lal Rawal, Nickolas M Reinig, Andre M Renzaho, Aziz L Rezapour, Alan L Robin, Luca Rossetti, Siamak Sabour, Sare Safi, Amirhossein Sahebkar, Mohammad Ali M Sahraian, Abdallah M Samy, Brijesh Sathian, Ganesh Kumar Saya, Mete A Saylan, Amira A Ali Shaheen, Masood Ali T Shaikh, Tueng T Shen, Kenji Shibabaw Shibuya, Wondimeneh Shibabaw Shiferaw, Mika Shigematsu, Jae Il Shin, Juan Carlos Silva, Alexander A Silvester, Jasvinder A Singh, Deepika S Singhal, Rita S Sitorus, Eirini Yurievich Skiadaresi, Valentin Yurievich Aleksandrovna Skryabin, Anna Aleksandrovna Skryabina, Amin Bekele Soheili, Muluken Bekele A R C Sorrie, Raúl A R C T Sousa, Chandrashekhar T Sreeramareddy, Dwight Girma Stambolian, Eyayou Girma Tadesse, Nina Ismail Tahhan, Md Ismail Tareque, Fotis Xuan Topouzis, Bach Xuan Tran, Gebiyaw K Tsegaye, Miltiadis K Tsilimbaris, Rohit Varma, Gianni Virgili, Avina Thu Vongpradith, Giang Thu Vu, Ya Xing Wang, Ningli Hailay Wang, Abrha Hailay K Weldemariam, Sheila K Gebeyehu West, Temesgen Gebeyehu Y Wondmeneh, Tien Y Wong, Mehdi Yaseri, Naohiro Yonemoto, Chuanhua Sergeevich Yu, Mikhail Sergeevich Zastrozhin, Zhi-Jiang R Zhang, Stephanie R Zimsen, Serge Resnikoff, Theo Vos, Vision Loss Expert Group of the Global Burden of Disease Study

## Abstract

**Background:**

Many causes of vision impairment can be prevented or treated. With an ageing global population, the demands for eye health services are increasing. We estimated the prevalence and relative contribution of avoidable causes of blindness and vision impairment globally from 1990 to 2020. We aimed to compare the results with the World Health Assembly Global Action Plan (WHA GAP) target of a 25% global reduction from 2010 to 2019 in avoidable vision impairment, defined as cataract and undercorrected refractive error.

**Methods:**

We did a systematic review and meta-analysis of population-based surveys of eye disease from January, 1980, to October, 2018. We fitted hierarchical models to estimate prevalence (with 95% uncertainty intervals [UIs]) of moderate and severe vision impairment (MSVI; presenting visual acuity from <6/18 to 3/60) and blindness (<3/60 or less than 10° visual field around central fixation) by cause, age, region, and year. Because of data sparsity at younger ages, our analysis focused on adults aged 50 years and older.

**Findings:**

Global crude prevalence of avoidable vision impairment and blindness in adults aged 50 years and older did not change between 2010 and 2019 (percentage change −0·2% [95% UI −1·5 to 1·0]; 2019 prevalence 9·58 cases per 1000 people [95% IU 8·51 to 10·8], 2010 prevalence 96·0 cases per 1000 people [86·0 to 107·0]). Age-standardised prevalence of avoidable blindness decreased by −15·4% [–16·8 to −14·3], while avoidable MSVI showed no change (0·5% [–0·8 to 1·6]). However, the number of cases increased for both avoidable blindness (10·8% [8·9 to 12·4]) and MSVI (31·5% [30·0 to 33·1]). The leading global causes of blindness in those aged 50 years and older in 2020 were cataract (15·2 million cases [9% IU 12·7–18·0]), followed by glaucoma (3·6 million cases [2·8–4·4]), undercorrected refractive error (2·3 million cases [1·8–2·8]), age-related macular degeneration (1·8 million cases [1·3–2·4]), and diabetic retinopathy (0·86 million cases [0·59–1·23]). Leading causes of MSVI were undercorrected refractive error (86·1 million cases [74·2–101·0]) and cataract (78·8 million cases [67·2–91·4]).

**Interpretation:**

Results suggest eye care services contributed to the observed reduction of age-standardised rates of avoidable blindness but not of MSVI, and that the target in an ageing global population was not reached.

**Funding:**

Brien Holden Vision Institute, Fondation Théa, The Fred Hollows Foundation, Bill & Melinda Gates Foundation, Lions Clubs International Foundation, Sightsavers International, and University of Heidelberg.

## Introduction

With rising sociodemographic status and life expectancy, many countries around the world are seeing more people live into adulthood, increases in the average age of the population, and a shift in the disease burden towards non-communicable diseases and disabilities. Most of the principal causes of vision impairment, including cataract and undercorrected refractive error,^1^ are subject to this epidemiological transition^2^ and carry significant individual and societal costs.^3^, ^4^ Cataract surgery and the dispensing of spectacles are among the most cost-effective health-care interventions currently available.^5^, ^6^, ^7^ Addressing these reversible conditions by scaling up existing health-care systems to provide access to cataract surgery and spectacles is an important opportunity. To highlight this need, the WHO and the International Agency for Prevention of Blindness created an initiative in 1999 called “Vision 2020: The Right to Sight”. In 2013, the World Health Asssembly (WHA) launched a new plan, Towards universal eye health: a global action plan 2014–2019 (GAP).^8^ It set a global target: to achieve by 2019 a 25% reduction from the baseline of 2010 in prevalence of “avoidable”^8^ visual impairment, defined as the aggregated crude prevalence of cataract and undercorrected refractive error (presenting visual acuity <6/18).

Previously, the Vision Loss Expert Group (VLEG) reported the results of a systematic review of population-based studies that reported the prevalence of blindness and vision impairment dating from 1980. These studies were compiled in a continuously updated database called the Global Vision Database.^1^, ^9^ WHO used these estimates as the basis for their 2019 world report on vision, which focused on people-centred eye care as a means for health system strengthening,^10^ and cause-specific data by region for 2015 were made available online.

**Research in context**

**Evidence before this study**

The growing and ageing of populations have led to increasing numbers of individuals with moderate or worse vision impairment globally. These trends triggered WHO and the International Agency for the Prevention of Blindness to create an initiative in 1999 called “Vision 2020: The Right to Sight”. This initiative set a goal to eliminate avoidable blindness. Previous publications by the Vision Loss Expert Group, and by the Global Burden of Diseases, Injuries, and Risk Factors Study demonstrated that, in 2015, cataract and undercorrected refractive error were responsible for the majority of moderate or worse vision impairment, and case numbers continued to rise over time.

**Added value of this study**

This study updates global and regional estimates of causes of moderate and severe vision impairment and blindness through 2020. We examined age-adjusted and sex-adjusted differences in the contribution of these causes to vision impairment, with a focus on older age groups. We incorporated studies from an updated systematic review for a total of 376 cause-specific sources. Rapid Assessment of Avoidable Blindness studies—key sources of vision loss data from low-income and middle-income settings—were disaggregated from prevalence for ages 50–99 years into 5-year age groups, providing more accurate data on age patterns. This update also allowed us to assess the World Health Assembly 2013 Global Action Plan (WHA GAP) target to reduce avoidable vision impairment, which was specifically defined as a reduction in moderate or worse vision impairment from undercorrected refractive error and cataract by 25% between 2010 and 2019.

**Implications of all the available evidence**

We found that in adults aged 50 years and older there was no change in the crude prevalence of avoidable vision impairment between 2010 and 2019, and case numbers increased. Cataract remained the largest contributor to global blindness in adults aged 50 years and older in 2020, with over 15 million individuals, approximately 45% of the 33·6 million cases of global blindness. Undercorrected refractive error remains the largest contributor to global moderate and severe vision impairment (MSVI) in adults aged 50 years and older, with over 86 million individuals, approximately 42% of the 206 million cases of global MSVI. Although less easily treatable, glaucoma, diabetic retinopathy, and age-related macular degeneration collectively led to more than 19 million cases of moderate or worse vision impairment in adults aged 50 years and older in 2020, making these diseases important targets for prevention and treatment. Age-standardised prevalence was higher in women than in men for all modelled causes of moderate or worse vision impairment, with the exception of glaucoma for which age-standardised prevalence was higher in men. Although the number of affected individuals increased for blindness due to all modelled causes, age-standardised prevalence for all modelled causes of vision except diabetic retinopathy has decreased over the past three decades. This suggests that eye care services did successfully reduce age-standardised prevalence, but they did not meet the growing need due to ageing and growth of the populations.

Since then, the VLEG has conducted a major update of the Global Vision Database in collaboration with researchers from the Global Burden of Diseases, Injuries, and Risk Factors Study (GBD study). Updated estimates are of particular interest because of recent rapid socioeconomic development, for example in China and south Asia. Additionally, the progressive emergence of causes of vision impairment such as myopic macular degeneration (particularly in China^11^, ^12^) and diabetic retinopathy as significant contributors to the vision impairment burden warrants a global update. With ageing populations, it was anticipated that two other conditions, glaucoma^13^ and age-related macular degeneration,^14^ would continue to be major causes of vision impairment. We now have more detailed and representative data sources from surveys of eye disease, bolstering our ability to track changes over time in cataract, undercorrected refractive error, macular degeneration, diabetic retinopathy, and glaucoma.

We report global and regional estimates of the burden of moderate and severe vision impairment (MSVI) and blindness due to cataract, undercorrected refractive error, glaucoma, age-related macular degeneration, and diabetic retinopathy. We examined temporal, sex, and age trends, with a focus on older age groups. We also assessed progress against the WHA GAP target of 25% reduction in avoidable vision impairment between 2010 and 2019.

## Methods

Estimates described here were produced in compliance with the Guidelines for Accurate and Transparent Health Estimates Reporting.^15^

### Input data

Preparation of data included first a systematic review of published population-based studies of vision impairment and blindness by the VLEG, that also included grey literature sources. Eligible studies from this review were then combined with data from Rapid Assessment of Avoidable Blindness (RAAB) studies by VLEG and finally data from the US National Health and Nutrition Examination survey and the WHO Study on Global Ageing and Adult Health were contributed by the GBD team. These stages are explained in more detail as follows. We included population-representative studies as data sources for cause-specific vision impairment modelling, primarily national and subnational cross-sectional surveys. RAAB surveys, which sample individuals aged 50 years and older, were major sources of data for low-income and middle-income settings.

The VLEG has systematically reviewed scientific literature published between 1980 and 2018 by commissioning the York Health Economics Consortium, UK, to search Embase, SciELO, MEDLINE, WHOLIS, and Open Grey, and additional grey literature sources. After title and abstract screening, abstracts were sent to regional committees of VLEG members to assess quality and make final inclusion decisions on whether to admit data to VLEG's Global Vision Database. Additionally, the VLEG commissioned the preparation of 5-year age-disaggregated RAAB data from the RAAB repository.

To meet inclusion criteria, visual acuity data had to be measured using a vision chart that could be mapped to the Snellen scale; studies based on self-report of vision impairment were excluded. We included studies that measured either presenting vision impairment (where visual acuity was measured using the usual corrective lenses a person arrived wearing), or best-corrected vision impairment (where a pinhole or lenses with power based on refraction were used to address any refractive error), or both. We applied WHO criteria for vision impairment severity, categorising people according to vision in the better-seeing eye on presentation. The categories were moderate vision impairment (defined as visual acuity of ≥6/60 and <6/18), severe vision impairment (visual acuity of ≥3/60 and <6/60), and blindness (visual acuity of <3/60 or <10° visual field around central fixation, although the visual field definition is rarely utilised in population-based eye surveys). We report a composite term of MSVI that comprises people meeting either moderate or severe visual acuity definitions. We also report a composite term of moderate or worse vision impairment that comprises people meeting moderate, severe, or blind visual acuity definitions.

### Data preparation

The separation of raw data into datasets, including total all-cause moderate vision impairment, severe vision impairment, and blindness, has been explained in detail elsewhere, in addition to a full list of the data sources.^16^ Cause-specific analyses are described below.

Presenting vision impairment was the reference definition for each level of severity. Undercorrected refractive error data were extracted directly from data sources where available, and otherwise calculated by subtracting best-corrected vision impairment from presenting vision impairment prevalence for each level of severity in studies that reported both measures for a given location, sex, age group, and year. All other causes were quantified as part of the best-corrected estimates of vision impairment at each level of severity.

We modelled distance vision impairment and blindness due to the following causes: cataract, undercorrected refractive error, age-related macular degeneration, myopic macular degeneration, glaucoma, diabetic retinopathy, and other causes of vision impairment (in aggregate). Minimum age for inclusion of data for these causes was set at 20 years for cataract and diabetic retinopathy; and 45 years for glaucoma and age-related macular degeneration. Other vision impairment estimates were combined with less prevalent causes of vision impairment to create a residual category (eg, retinopathy of prematurity, vitamin A deficiency, trachoma). Trachoma data were extracted as a proportion, in which the numerator was total cases of trachoma, and the denominator was total cases of vision impairment at a given severity level. Geographic restrictions were applied so that zero prevalence was imputed for non-endemic locations.

Data collected using RAAB methodology were adjusted to comprehensive surveys (reference definition) using the same adjustment factors as for all-cause MSVI and blindness (described in a sister paper on all-cause vision loss).^16^ This approach was used because there were many more data available for all-cause moderate vision impairment, severe vision impairment, and blindness than for each specific cause, allowing for more data-rich models.

### Disease modelling meta-regression 2.1 modelling

We produced location, year, age, and sex-specific estimates of MSVI and blindness using Disease Modelling Meta-Regression (Dismod-MR) 2.1, which is described in detail elsewhere.^16^, ^17^, ^18^ Global estimates are first produced with a mixed effects non-linear model using all available data to produce a global model fit as well as fixed and random effects. These outputs are passed to the GBD super-region level (seven super-regions) as a prior, and super-region fits are generated using super-region input data and the global prior. The super-region outputs are passed to the region level (21 regions), then to the country level, and finally the subnational level for 21 countries. Final estimates were generated by aggregation, for which the region final was the sum of country estimates, etc. To location aggregate (eg, super-regions or global estimates), we aggregated the most granular estimates using straightforward summation of cases, which were then used to compute all-age and age-standardised rates. For location estimates, we used draw-level regional estimates of regional case numbers (“draws” from the 1000 posterior runs of the model) and summed these to obtain super-region and global case numbers. We then summarised the draw level estimates by taking the mean across values. No weighting was used in the global or regional aggregations. For avoidable vision loss we took draw-level regional estimates of case numbers for uncorrected refractive error and cataract and summed these together.

During data processing, we applied data adjustments if we knew there were potential measurement errors using a meta-regression tool developed at Institute for Health Metrics and Evaluation (IHME) called MR-BRT.^17^ For example, our reference definition for all-cause vision loss was presenting visual acuity systematically on the basis of the results of a regression analysis comparing the prevalence of the two methods. We similarly adjusted for studies that used RAAB methodology or non-standard severity definitions. Our estimation tool (Dismod) made quantification of between-study heterogeneity—the part of variance not ascribed to fixed effects or geographical random effects–and added uncertainty based on that finding.

### Modelling and post-processing steps

Dismod-MR 2.1 models were run for all vision impairment by severity (moderate, severe, blindness) regardless of cause and, separately, for MSVI and blindness due to each modelled cause of vision impairment (eg, MSVI due to cataract and blindness due to cataract). Then, models of MSVI due to specific causes were split into moderate and severe estimates using the ratio of overall prevalence in the all-cause moderate presenting vision impairment and severe presenting vision impairment models. Next, prevalence estimates for all causes by severity were scaled to the models of all-cause prevalence by severity. This produced final estimates by age, sex, year, and location for each individual cause of vision impairment by severity. Models were iterated until reaching convergence, and estimates were calculated with the final 1000 model runs, or draws. These draws were used to produce mean estimates and 95% uncertainty intervals (UIs), bounded by the 25th and 975th values of the ordered 1000 draws.

To assess whether targets of the WHA GAP were reached in the 2010–19 period, we aggregated estimates for cataract and undercorrected refractive error to create the “avoidable vision impairment” category used as a metric for assessing whether the GAP target was met.

### Role of the funding source

The funder of the study had no role in study design, data collection, data analysis, data interpretation, or writing of the report. All authors had access to all estimates presented in the paper, and the corresponding author had final responsibility for the decision to submit for publication.

## Results

We used 512 data sources to calculate the prevalence of categories of distance vision impairment. 376 data sources reported cause-specific data disaggregated to include at least one of the following: undercorrected refractive error, cataract, glaucoma, age-related macular degeneration, myopic macular degeneration, or diabetic retinopathy (appendix p 7). 230 (61%) of these 376 data sources were RAABs.^16^ Many studies incorporated in the 2017 update^1^ have since submitted more granular levels of disaggregated data, enabling more precise estimates of the causes of global vision impairment in 2020 and their temporal changes. Data sources for blindness and MSVI caused by myopic macular degeneration were sparse globally with the majority of sources from China. For this reason, we reported estimates of myopic macular degeneration solely for China. Globally, data for children and young adults were also sparse, as were data for high-income locations. Because of the data sparsity at younger ages, we focused our analyses on adults aged 50 years and older.

To assess the success of the WHA GAP to reduce avoidable vision impairment, we calculated the change in avoidable vision impairment over the past decade. Crude prevalence of all moderate or worse avoidable vision impairment between 2010 and 2019 in adults aged 50 years and older did not change (percentage change of −0·2% [95% UI −1·5 to 1·0]); 2019 prevalence 95·8 cases per 1000 people [85·1 to 108·0], 2010 prevalence 96·0 cases per 1000 people [86·0 to 107·]), but it increased in all ages by 9·1% (7·6 to 10·7). Total cases of moderate or worse avoidable vision impairment increased in people aged 50 years and older by 29·2% (27·6 to 30·9) and in all ages by 20·8% (19·2 to 22·6) for a composite 211 million cases of moderate or worse vision impairment in 2010 to 254 million cases in 2019. Crude prevalence by cause and severity in 2020 is given in the appendix (pp 4–5).

Underlying the overall lack of change in crude prevalence of all moderate or worse avoidable vision impairment in people aged 50 years and older, avoidable MSVI showed little change between 2010 and 2019, with an increase of 1·6% (0·4 to 2·8), whereas avoidable blindness decreased by −14·4% (–15·9 to −13·2; table 1). Similarly, age-standardised prevalence of avoidable MSVI showed no change (0·5% [–0·8 to 1·6]), but blindness decreased (–15·4% [–16·8 to −14·3]). By contrast, the number of cases increased markedly for both avoidable MSVI (31·5% [30·0 to 33·1]) and avoidable blindness (10·8% [8·9 to 12·4]).

**Table 1:** Percentage change in crude prevalence of moderate and severe vision impairment and blindness in adults aged 50 years and older between 2010 and 2019

	**Moderate and severe vision impairment**			**Blindness**		
	Crude prevalence	Number of cases	Age-standardised prevalence	Crude prevalence	Number of cases	Age-standardised prevalence
All causes	1·9% (0·8 to 3·1)	32·0% (30·5 to 33·5)	0·7% (−0·5 to 1·8)	−10·0% (−11·2 to −9·1)	16·5% (15·0 to 17·8)	−11·4% (−12·3 to −10·6)
Avoidable* (cataract+ undercorrected refractive error)	1·6% (0·4 to 2·8)	31·5% (30·0 to 33·1)	0·5% (−0·8 to 1·6)	−14·4% (−15·9 to −13·2)	10·8% (8·9 to 12·4)	−15·4% (−16·8 to −14·3)
Cataract	2·9% (1·5 to 4·2)	33·2% (31·4 to 34·9)	1·1% (−0·3 to 2·3)	−14·0% (−15·5 to −12·7)	11·4% (9·5 to 13·1)	−15·1% (−16·4 to −13·9)
Undercorrected refractive error	0·4% (−1·1 to 1·9)	30·0% (28·1 to 31·9)	−0·1% (−1·5 to 1·3)	−17·5% (−19·2 to −15·7)	6·9% (4·6 to 9·1)	−17·8% (−19·5 to −16·3)
Glaucoma	10·7% (9·2 to 12·3)	43·4% (41·4 to 45·5)	8·0% (6·5 to 9·5)	−8·3% (−9·8 to −7·0)	18·7% (16·9 to 20·4)	−10·8% (−11·9 to −9·9)
Age-related macular degeneration	7·1% (5·2 to 9·0)	38·7% (36·2 to 41·2)	5·0% (3·2 to 6·7)	−9·1% (−11·4 to −7·0)	17·7% (14·8 to 20·4)	−11·7% (−13·8 to −9·8)
Diabetic retinopathy	0·8% (−0·9 to 2·6)	30·6% (28·3 to 32·8)	−0·4% (−2·1 to 1·2)	−6·0% (−8·4 to −3·5)	21·8% (18·7 to 25·0)	−6·6% (−9·0 to −4·1)
Residual causes of vision loss	1·8% (0·7 to 3·1)	31·9% (30·4 to 33·5)	0·0% (−0·9 to 1·1)	−2·4% (−4·1 to −0·8)	26·4% (24·2 to 28·5)	−3·7% (−5·2 to −2·3)

Data in parentheses are 95% uncertainty intervals. Data for all ages are given in the appendix (p 6). Data presented in this table were calculated to assess the success of the World Health Assembly's global action plan to reduce avoidable vision impairment.

* Classified as “avoidable” in the global action plan.

We then looked at the contribution of individual causes to global visual impairment in 2020. Among the global 33·6 million adults aged 50 years and older who were blind in 2020^16^ the leading causes of blindness (table 2) were cataract (15·2 million cases [95% UI 12·7–18·0]), followed by glaucoma (3·6 million cases [2·8–4·4]), undercorrected refractive error (2·3 million cases [1·8–2·8]), age-related macular degeneration (1·8 million cases [1·3–2·4]), and diabetic retinopathy (0·9 million cases [0·6–1·2]). For the estimated 206 million aged 50 years and older adults with MSVI in 2020,^16^ the leading causes of MSVI (table 3) were undercorrected refractive error (86·1 million cases [74·2–101·0]), followed by cataract (78·8 million cases [67·2–91·4]), age-related macular degeneration (6·2 million cases [5·0–7·6]), glaucoma (4·1 million cases [3·2–5·2]), and diabetic retinopathy (2·9 million cases [2·1–3·9]).

**Table 2:** Cases and age-standardised prevalence in 2020 for blindness and percentage changes from 1990 to 2020 in adults aged 50 years and older, by cause of blindness

	**Cases (thousands)**		**Age-standardised prevalence (per 1000)**	
	2020	Percentage change from 1990 to 2020	2020	Percentage change from 1990 to 2020
**Cataract**				
Global	15 200 (12700 to 18000)	55·7% (51·4 to 59·9)	8·38 (7·04 to 9·93)	−31·7% (−33·2 to −30·1)
Central Europe, eastern Europe, and central Asia	266 (211 to 332)	−4·3% (−7·3 to −1·4)	1·88 (1·50 to 2·33)	−36·1% (−37·5 to −34·6)
High income	456 (367 to 566)	56·0% (48·2 to 64·6)	0·877 (0·702 to 1·08)	−22·6% (−24·2 to −21·3)
Latin America and Caribbean	1010 (826 to 1210)	78·4% (73·2 to 83·7)	7·85 (6·42 to 9·48)	−43·8% (−44·7 to −42·7)
North Africa and Middle East	756 (593 to 941)	30·0% (24·9 to 35·4)	9·06 (7·13 to 11·4)	−53·7% (−55·3 to −52·0)
South Asia	5910 (4990 to 6970)	51·1% (44·7 to 57·6)	22·3 (18·9 to 26·1)	−46·4% (−48·1 to −44·5)
Southeast Asia, east Asia, and Oceania	5540 (4620 to 6590)	67·2% (62·5 to 72·5)	9·72 (8·14 to 11·5)	−43·8% (−45·6 to −41·6)
Sub-Saharan Africa	1240 (1030 to 1490)	54·4% (51·3 to 57·7)	14·9 (12·4 to 17·8)	−30·5% (−31·7 to −29·2)
**Undercorrected refractive error**				
Global	2290 (1790 to 2800)	54·4% (48·7 to 60·6)	1·22 (0·960 to 1·50)	−28·7% (−31·1 to −26·0)
Central Europe, eastern Europe, and central Asia	16·8 (12·8 to 21·1)	9·0% (5·7 to 12·0)	0·121 (0·0912 to 0·150)	−17·0% (−19·2 to −14·6)
High income	46·1 (35·7 to 56·9)	35·1% (29·9 to 40·5)	0·103 (0·079 to 0·128)	−23·1% (−24·8 to −21·4)
Latin America and Caribbean	126 (97·5 to 153)	97·3% (91·3 to 103·0)	0·943 (0·731 to 1·14)	−31·2% (−32·9 to −29·6)
North Africa and Middle East	84·2 (63·8 to 103)	68·3% (61·5 to 74·7)	0·875 (0·673 to 1·07)	−36·1% (−38·5 to −33·9)
South Asia	976 (762 to 1190)	25·8% (19·9 to 32·0)	3·30 (2·59 to 4·00)	−52·3% (−54·0 to −50·5)
Southeast Asia, east Asia, and Oceania	933 (728 to 1140)	91·5% (82·8 to 101·5)	1·47 (1·16 to 1·79)	−27·9% (−31·0 to −24·3)
Sub-Saharan Africa	111 (84·5 to 136)	91·4% (85·3 to 98·0)	1·14 (0·881 to 1·41)	−15·0% (−17·2 to −12·8)
**Glaucoma**				
Global	3600 (2800 to 4410)	61·8% (57·2 to 66·8)	2·04 (1·59 to 2·49)	−31·9% (−33·0 to −30·6)
Central Europe, eastern Europe, and central Asia	178 (139 to 219)	5·5% (1·9 to 9·3)	1·25 (0·972 to 1·53)	−31·4% (−32·8 to −30·0)
High income	785 (622 to 964)	60·3% (53·0 to 69·0)	1·41 (1·12 to 1·74)	−23·2% (−24·5 to −21·9)
Latin America and Caribbean	334 (256 to 411)	105·5% (98·4 to 113·6)	2·63 (2·01 to 3·23)	−36·2% (−37·6 to −34·8)
North Africa and Middle East	463 (354 to 578)	64·8% (57·6 to 72·3)	5·69 (4·37 to 7·10)	−40·9% (−43·0 to −39)
South Asia	577 (439 to 726)	75·7% (65·1 to 86·7)	2·26 (1·71 to 2·83)	−38·7% (−41·0 to −36·1)
Southeast Asia, east Asia, and Oceania	754 (575 to 957)	52·4% (44·5 to 61·3)	1·34 (1·02 to 1·67)	−48·6% (−51·3 to −45·6)
Sub-Saharan Africa	510 (398 to 628)	70·1% (65·1 to 75·2)	6·64 (5·20 to 8·09)	−23·5% (−25·5 to −21·5)
**Age-related macular degeneration**				
Global	1840 (1340 to 2420)	69·8% (64·4 to 75·3)	1·03 (0·755 to 1·36)	−28·0% (−30·0 to −25·6)
Central Europe, eastern Europe, and central Asia	62·5 (43·2 to 84·0)	21·8% (17·2 to 27·5)	0·437 (0·305 to 0·587)	−16·8% (−18·5 to −14·9)
High income	595 (455 to 768)	48·3% (40·8 to 56·3)	1·08 (0·828 to 1·39)	−28·6% (−30·0 to −27·3)
Latin America and Caribbean	71·1 (49·1 to 97·1)	142·1% (131·5 to 152·5)	0·550 (0·379 to 0·747)	−21·2% (−23·3 to −18·8)
North Africa and Middle East	194 (136 to 264)	105·1% (95·0 to 115·8)	2·23 (1·55 to 2·99)	−23·3% (−26·5 to −20·1)
South Asia	296 (199 to 421)	53·4% (42·4 to 65·4)	1·05 (0·717 to 1·47)	−41·0% (−44·1 to −37·5)
Southeast Asia, east Asia, and Oceania	492 (340 to 673)	104·0% (91·8 to 116·5)	0·835 (0·578 to 1·14)	−27·3% (−31·3 to −22·9)
Sub-Saharan Africa	130 (91·4 to 178)	78·5% (70·2 to 86·5)	1·50 (1·05 to 2·04)	−19·1% (−23·0 to −15·7)
**Diabetic retinopathy**				
Global	861 (592 to 1230)	150·9% (143·3 to 159·0)	0·459 (0·316 to 0·658)	14·9% (11·4 to 18·4)
Central Europe, eastern Europe, and central Asia	11·9 (7·96 to 17·3)	26·6% (17·8 to 36·4)	0·0842 (0·0565 to 0·121)	−6·1% (−11·8 to −0·2)
High income	139 (97·2 to 196)	53·2% (44·2 to 62·2)	0·307 (0·215 to 0·433)	−13·7% (−17·6 to −9·7)
Latin America and Caribbean	203 (142 to 283)	130·7% (121·7 to 140·5)	1·50 (1·05 to 2·09)	−17·9% (−20·5 to −15·2)
North Africa and Middle East	61·0 (40·1 to 91·8)	169·3% (149·2 to 193·1)	0·621 (0·413 to 0·928)	0·9% (−6·2 to 9·3)
South Asia	152 (101 to 225)	190·7% (162·6 to 222·9)	0·487 (0·330 to 0·708)	17·9% (7·8 to 30·1)
Southeast Asia, east Asia, and Oceania	261 (171 to 387)	286·7% (257·9 to 316·2)	0·406 (0·269 to 0·602)	44·5% (33·1 to 56·3)
Sub-Saharan Africa	33·9 (23·0 to 49·2)	177·4% (162·4 to 192·3)	0·341 (0·234 to 0·498)	25·7% (19·4 to 32·3)
**Residual causes of vision loss**				
Global	9840 (8200 to 11500)	69·3% (62·0 to 76·5)	5·33 (4·46 to 6·24)	−24·2% (−27·3 to −21·3)
Central Europe, eastern Europe, and central Asia	647 (545 to 754)	11·7% (8·7 to 14·9)	4·63 (3·92 to 5·38)	−22·5% (−23·9 to −21·1)
High-income	551 (461 to 650)	48·5% (43·1 to 54·9)	1·22 (1·03 to 1·44)	−16·5% (−18·5 to −14·4)
Latin America and Caribbean	1140 (940 to 1350)	125·2% (117·5 to 133·1)	8·69 (7·16 to 10·3)	−25·8% (−27·6 to −24·1)
North Africa and Middle East	788 (631 to 959)	73·7% (65·1 to 82·5)	8·48 (6·79 to 10·4)	−35·6% (−38·8 to −32·8)
South Asia	1670 (1370 to 2010)	23·4% (12·1 to 36·5)	5·93 (4·84 to 7·10)	−54·2% (−58·5 to −49·7)
Southeast Asia, east Asia, and Oceania	3850 (3230 to 4500)	117·0% (108·1 to 125·6)	6·35 (5·33 to 7·40)	−23·0% (−26·2 to −19·8)
Sub-Saharan Africa	1200 (973 to 1440)	54·7% (49·4 to 60·0)	12·9 (10·4 to 15·3)	−30·0% (−32·1 to −27·8)

Data in parentheses are 95% uncertainty intervals. Data for all ages are given in the appendix (p 2).

**Table 3:** Cases and age-standardised prevalence in 2020 for moderate and severe vision impairment and percent changes from 1990 to 2020 in adults aged 50 years and older, by cause of vision impairment

	**Cases (thousands)**		**Age-standardised prevalence (per 1000)**	
	2020	Percentage change from 1990 to 2020	2020	Percentage change from 1990 to 2020
**Cataract**				
Global	78 800 (67200 to 91400)	175·2% (170·9 to 179·5)	43·4 (37·1 to 50·2)	19·2% (17·8 to 20·5)
Central Europe, eastern Europe, and central Asia	3050 (2490 to 3620)	49·7% (46·2 to 53·0)	21·3 (17·5 to 25·2)	0·4% (−1·1 to 1·9)
High income	7880 (6660 to 9230)	100·2% (94·0 to 106·5)	14·6 (12·2 to 17·1)	−1·8% (−2·8 to −0·7)
Latin America and Caribbean	4350 (3650 to 5090)	208·6% (201·8 to 215·7)	33·9 (28·5 to 39·6)	−1·2% (−2·4 to 0·14)
North Africa and Middle East	5020 (4230 to 5920)	181·0% (172·6 to 189·6)	58·1 (49·2 to 68·0)	0·6% (−2·1 to 3·6)
South Asia	27200 (23200 to 31800)	180·7% (171·8 to 189·6)	94·6 (81·1 to 109)	1·7% (−0·3 to 3·6)
Southeast Asia, east Asia, and Oceania	26 800 (23000 to 30900)	235·5% (228·3 to 242·7)	47·1 (40·4 to 54·1)	13·5% (11·7 to 15·3)
Sub-Saharan Africa	4440 (3780 to 5160)	150·8% (146·1 to 156·1)	51·4 (44·0 to 59·3)	11·4% (9·7 to 13·4)
**Undercorrected refractive error**				
Global	86 100 (74200 to 101000)	101·8% (98·9 to 104·9)	45·8 (39·6 to 53·7)	−6·9% (−8·0 to −5·9)
Central Europe, eastern Europe, and central Asia	6340 (5400 to 7480)	25·8% (23·2 to 28·3)	45·1 (38·5 to 53·1)	−4·4% (−5·5 to −3·3)
High income	8940 (7680 to 10400)	69·6% (66·3 to 72·9)	19·4 (16·7 to 22·5)	−4·6% (−5·6 to −3·6)
Latin America and Caribbean	5780 (4950 to 6780)	162·3% (158·5 to 165·8)	42·8 (36·8 to 50·0)	−7·7% (−8·8 to −6·6)
North Africa and Middle East	4680 (3960 to 5550)	140·5% (134·2 to 147·2)	47·3 (40·2 to 55·4)	−10·7% (−13·2 to −8·0)
South Asia	32 100 (27500 to 37900)	94·1% (89·6 to 99·7)	103 (88·2 to 121)	−23·4% (−24·9 to −21·9)
Southeast Asia, east Asia, and Oceania	25 000 (21500 to 29300)	143·7% (138·4 to 148·7)	39·4 (33·9 to 45·6)	−8·8% (−10·6 to −7·1)
Sub-Saharan Africa	3210 (2730 to 3800)	131·7% (126·7 to 136·4)	31·6 (27·3 to 37·0)	2·4% (0·6 to 4·1)
**Glaucoma**				
Global	4130 (3240 to 5170)	151·2% (147·2 to 155·3)	2·29 (1·80 to 2·86)	8·3% (6·8 to 9·9)
Central Europe, eastern Europe, and central Asia	213 (167 to 270)	43·6% (40·0 to 47·6)	1·47 (1·15 to 1·86)	−3·0% (−4·7 to −1·4)
High income	596 (467 to 762)	103·2% (95·7 to 111·5)	1·09 (0·853 to 1·39)	−0·2% (−1·5 to 1·0)
Latin America and Caribbean	498 (390 to 623)	191·7% (185·2 to 199·2)	3·86 (3·02 to 4·84)	−4·7% (−6·1 to −3·1)
North Africa and Middle East	325 (251 to 419)	148·1% (139·1 to 157·7)	3·76 (2·87 to 4·85)	−9·7% (−12·8 to −6·5)
South Asia	952 (745 to 1200)	145·2% (136·4 to 154·5)	3·38 (2·66 to 4·21)	−12·8% (−15·0 to −10·6)
Southeast Asia, east Asia, and Oceania	1160 (916 to 1450)	251·5% (242·0 to 262·0)	2·01 (1·58 to 2·52)	21·4% (18·3 to 24·8)
Sub-Saharan Africa	391 (306 to 493)	111·9% (107·1 to 116·8)	4·57 (3·61 to 5·69)	−4·3% (−6·3 to −2·2)
**Age-related macular degeneration**				
Global	6220 (5030 to 7570)	150·2% (145·9 to 154·8)	3·39 (2·75 to 4·12)	10·6% (8·7 to 12·6)
Central Europe, eastern Europe, and central Asia	228 (182 to 282)	51·1% (47·8 to 54·3)	1·57 (1·26 to 1·95)	4·8% (3·13 to 6·51)
High income	738 (584 to 917)	76·8% (70·5 to 83·3)	1·39 (1·11 to 1·72)	−9·4% (−11·2 to −7·5)
Latin America and Caribbean	333 (269 to 407)	202·2% (193·0 to 212·2)	2·55 (2·07 to 3·13)	1·5% (−1·2 to 4·5)
North Africa and Middle East	493 (390 to 613)	166·0% (156·5 to 175·6)	5·48 (4·36 to 6·77)	−0·5% (−4·0 to 2·8)
South Asia	1220 (968 to 1510)	121·4% (112·8 to 130·2)	4·18 (3·36 to 5·11)	−20·5% (−23·0 to −18·0)
Southeast Asia, east Asia, and Oceania	2760 (2210 to 3380)	214·6% (204·7 to 224·4)	4·61 (3·73 to 5·61)	13·8% (10·7 to 17·1)
Sub-Saharan Africa	453 (359 to 565)	132·2% (124·6 to 140·3)	4·96 (3·98 to 6·14)	5·6% (2·3 to 9·0)
**Diabetic retinopathy**				
Global	2950 (2140 to 3950)	129·5% (123·2 to 135·9)	1·59 (1·15 to 2·12)	3·3% (0·4 to 5·8)
Central Europe, eastern Europe, and central Asia	134 (94·2 to 183)	21·7% (17·3 to 25·9)	0·942 (0·661 to 1·28)	−11·3% (−13·9 to −8·7)
High income	386 (277 to 522)	68·4% (63·5 to 74·1)	0·802 (0·578 to 1·08)	−7·7% (−9·7 to −6·0)
Latin America and Caribbean	396 (290 to 533)	185·6% (177·6 to 193·6)	2·97 (2·17 to 4·01)	−0·9% (−3·1 to 1·4)
North Africa and Middle East	399 (288 to 540)	115·9% (106·3 to 125·6)	4·14 (3·00 to 5·53)	−19·6% (−23·2 to −16·0)
South Asia	389 (279 to 527)	123·7% (114·5 to 134·5)	1·28 (0·921 to 1·72)	−15·8% (−19·2 to −12·4)
Southeast Asia, east Asia, and Oceania	1100 (796 to 1500)	182·7% (166·3 to 200·2)	1·79 (1·29 to 2·42)	3·2% (−2·6 to 9·0)
Sub-Saharan Africa	138 (98·6 to 186)	145·3% (138·2 to 152·4)	1·44 (1·04 to 1·92)	9·9% (7·1 to 12·8)
**Residual causes of vision loss**				
Global	28 200 (24100 to 33000)	104·4% (100·3 to 108·5)	15·2 (13·0 to 17·8)	−8·3% (−9·7 to −6·9)
Central Europe, eastern Europe, and central Asia	4180 (3540 to 4950)	34·6% (31·9 to 37·5)	29·1 (24·7 to 34·3)	−5·7% (−7·0 to −4·4)
High income	3060 (2570 to 3630)	82·6% (77·6 to 88·5)	6·32 (5·34 to 7·50)	−1·6% (−2·9 to −0·3)
Latin America and Caribbean	2710 (2260 to 3230)	176·9% (171·1 to 183·2)	20·5 (17·1 to 24·3)	−7·6% (−8·9 to −6·3)
North Africa and Middle East	1090 (890 to 1330)	112·1% (98·8 to 124·5)	11·6 (9·40 to 14·1)	−22·1% (−26·7 to −17·6)
South Asia	6900 (5780 to 8250)	141·1% (132·4 to 151·1)	22·9 (19·5 to 27·0)	−5·7% (−8·5 to −2·8)
Southeast Asia, east Asia, and Oceania	7850 (6780 to 9100)	158·1% (148·7 to 167·5)	12·7 (11·0 to 14·7)	−5·9% (−8·7 to −3·1)
Sub-Saharan Africa	2410 (2020 to 2880)	49·0% (42·0 to 56·0)	24·7 (20·8 to 29·2)	−32·4% (−35·5 to −29·3)

Data in parentheses are 95% uncertainty intervals. Data for all ages are given in the appendix (p 3).

In terms of relative contribution to age-standardised prevalence of total blindness in adults aged 50 years and older (table 4), cataract caused 45·5% (41·7–49·0) of all global blindness, followed by glaucoma (11·0% [9·3–12·8]), undercorrected refractive error (6·6% [5·6–7·8]), age-related macular degeneration (5·6% [4·3–7·0]) and diabetic retinopathy (2·5% [1·7–3·7]). The leading contributor to global age-standardised prevalence of adult MSVI was undercorrected refractive error (table 4; 41·0% [38·0–44·1]), followed by cataract (38·9% [35·6–42·4]), age-related macular degeneration (3·0% [2·5–3·5]), glaucoma (2·1% [1·7–2·5]), and diabetic retinopathy (1·4% [1·0–1·9]).

**Table 4:** Relative percentage contribution of each cause to age-standardised prevalence of blindness and moderate and severe vision impairment by superregion in 2020 in adults aged 50 years and older

	**Cataract**	**Undercorrected refractive error**	**Glaucoma**	**Age-related macular degeneration**	**Diabetic retinopathy**	**Residual causes of vision loss**
**Blindness**						
Global	45·4% (41·7–49·0)	6·6% (5·6–7·8)	11% (9·3–12·8)	5·6% (4·3–6·7)	2·5% (1·7–3·7)	28·9% (26·5–31·5)
Central Europe, eastern Europe, and central Asia	22·4% (19·1–25·9)	1·4% (1·2–1·8)	14·8% (12·3–17·3)	5·2% (3·8–6·7)	1·0% (0·7–1·5)	55·1% (51·0–59·6)
High income	17·5% (14·9–20·5)	2·1% (1·7–2·5)	28·2% (24·0–32·3)	21·6% (17·5–26·1)	6·2% (4·2–8·8)	24·5% (21·6–27·8)
Latin America and Caribbean	35·4% (31·4–39·8)	4·3% (3·6–5·0)	11·8% (9·7–13·9)	2·5% (1·8–3·2)	6·8% (4·5–9·6)	39·2% (35·5–43·4)
North Africa and Middle East	33·6% (29·4–38·0)	3·3% (2·7–3·8)	21·1% (17·7–24·5)	8·3% (6·2–10·5)	2·3% (1·5–3·6)	31·5% (27·8–36·0)
South Asia	63·1% (59·4–66·8)	9·4% (7·9–10·9)	6·4% (5·2–7·7)	3·0% (2·1–4·0)	1·4% (0·9–2·1)	16·8% (15·0–18·8)
Southeast Asia, east Asia, and Oceania	48·3% (44·5–52·1)	7·3% (6·2–8·5)	6·7% (5·5–7·9)	4·2% (3·0–5·4)	2·0% (1·3–3·0)	31·6% (29·0–34·3)
Sub-Saharan Africa	39·8% (35·9–43·9)	3·1% (2·6–3·6)	17·8% (15·1–20·6)	4·0% (3·0–5·1)	0·9% (0·6–1·4)	34·4% (31·4–37·7)
**Moderate and severe vision impairment**						
Global	38·9% (35·6–42·4)	41% (38·0–44·1)	2·1% (1·7–2·5)	3·0% (2·5–3·5)	1·4% (1·0–2·0)	13·6% (12·3–15·2)
Central Europe, eastern Europe, and central Asia	21·4% (18·8–24·2)	45·3% (42·0–48·6)	1·5% (1·2–1·8)	1·6% (1·3–1·9)	1·0% (0·7–1·3)	29·3% (26·2–32·5)
High income	33·5% (29·8–37·2)	44·5% (41·1–48·0)	2·5% (2·0–3·1)	3·2% (2·7–3·8)	1·9% (1·3–2·5)	14·5% (12·7–16·8)
Latin America and Caribbean	31·8% (28·4–35·3)	40·2% (37·1–43·4)	3·6% (3·0–4·4)	2·4% (2·0–2·8)	2·8% (2·0–3·8)	19·2% (17·0–21·8)
North Africa and Middle East	44·6% (40·6–48·8)	36·3% (32·7–39·8)	2·9% (2·3–3·6)	4·2% (3·5–5·0)	3·2% (2·3–4·3)	8·9% (7·6–10·6)
South Asia	41·3% (37·8–45)	44·9% (41·3–48·1)	1·5% (1·2–1·8)	1·8% (1·5–2·2)	0·6% (0·4–0·8)	10% (8·9–11·2)
Southeast Asia, east Asia, and Oceania	43·8% (40·4–47·2)	36·6% (33·7–39·7)	1·9% (1·5–2·3)	4·3% (3·6–5·0)	1·7% (1·2–2·3)	11·8% (10·8–13·0)
Sub-Saharan Africa	43·3% (39·8–46·8)	26·7% (24·4–29·0)	3·9% (3·2–4·8)	4·2% (3·5–4·9)	1·2% (0·9–1·6)	20·8% (18·7–23·0)

Data in parentheses are 95% uncertainty intervals.

**Figure 1:**
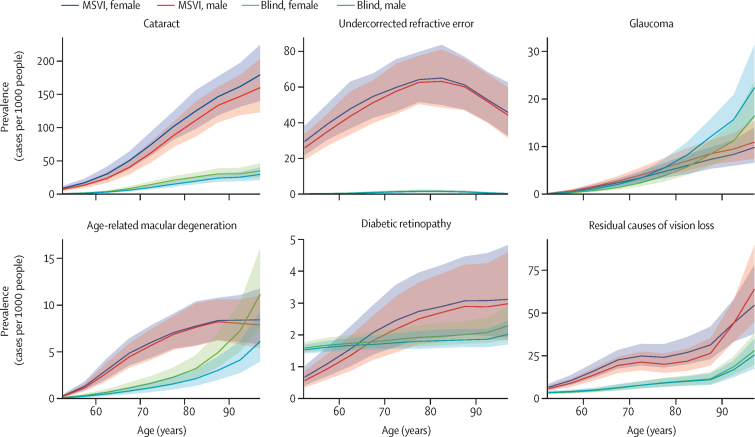
Age-specific prevalence rates of distance blindness and MSVI by cause and sex in adults aged 50 years and older in 2020 Solid lines show sex-specific prevalence estimates, with shaded areas indicating 95% uncertainty intervals. MSVI=moderate to severe vision impairment.

Overall, prevalence of vision impairment increased with age, although the pattern of age-specific prevalence varied by cause and severity (figure 1). MSVI prevalence was consistently higher than blindness for all causes, with the exception of glaucoma and age-related macular degeneration at the oldest ages. MSVI due to most causes increased from age 50 years onward, although MSVI due to undercorrected refractive error increased up to the age of around 80 years and decreased thereafter.

**Figure 2:**
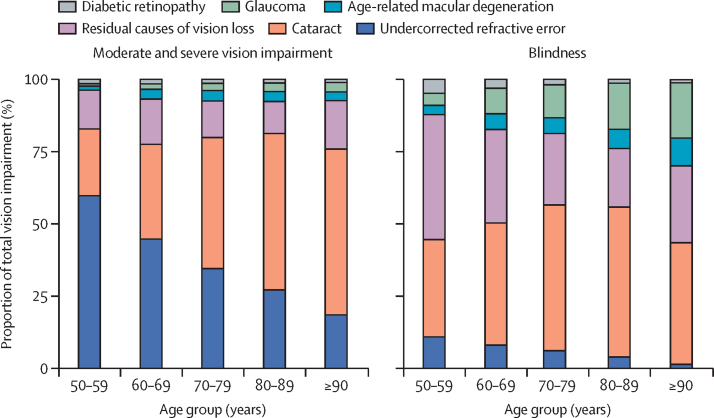
Relative contribution of each cause to crude prevalence of blindness and moderate and severe vision impairment in 2020 by age group

Of the explicitly modelled causes of global blindness, cataract was the principal cause of global blindness in all 10-year age groups for ages 50 years and older (figure 2). The contribution of glaucoma and age-related macular degeneration were greatest in the oldest age group, while the contributions of diabetic retinopathy to blindness decreased with age. For global MSVI, undercorrected refractive error was the principal contributor in the age groups 50–59 and 60–69 years, and cataract was the principal cause in those aged 70 years and older.

We compared age-standardised prevalence between men and women aged 50 years and older for each cause of vision impairment and found that age-standardised prevalence of blindness was greater in women for cataract (difference between means: 0·19% [0·16 to 0·24]), undercorrected refractive error (0·006% [0·003 to 0·01]), age-related macular degeneration (0·037% [0·028 to 0·047]), and diabetic retinopathy (0·008% [0·005 to 0·001]), and greater in men for glaucoma (–0·072 [–0·086 to −0·058]. The same patterns were found for sex differences in causes of MSVI (data not shown).

When looking at geographical trends, in 2020, cataract was the largest contributor to blindness in adults aged 50 years and older in all super-regions except for the high-income super-region, where the largest contributor was glaucoma (table 4). This was primarily driven by two regions, western Europe (glaucoma: 32·5% [27·3 to 37·3] *vs* cataract: 11·4% [9·4 to 34·9]) and high-income Asia Pacific (glaucoma: 33·7% [29·4 to 37·7] *vs* cataract: 20·5% [17·7 to 23·9]). For MSVI (table 4), cataract was the leading contributor in western and eastern sub-Saharan Africa, southeast Asia, Oceania, and north Africa and Middle East. Cataract and undercorrected refractive error contributed similarly in central Asia, Andean Latin America, east Asia, and south Asia; undercorrected refractive error was the largest contributor everywhere else.

**Figure 3:**
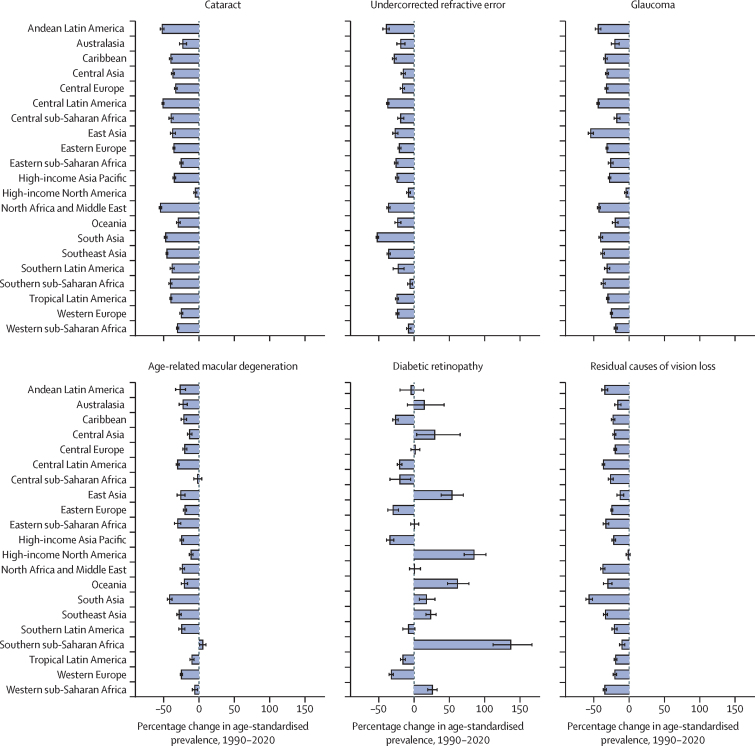
Change in regional age-standardised prevalence of blindness between 1990 and 2020 in adults aged 50 years and older, by cause of blindness Data for moderate and severe vision impairment are presented in appendix (p 8).

Age-standardised prevalence of blindness for all modelled causes consistently showed a regional decrease between 1990 and 2020 (figure 3). The notable exception to this trend was diabetic retinopathy, for which prevalence increased in many regions, with the largest increase in southern sub-Saharan Africa (percentage change 138·3% [113·0–168·5]). The pattern for MSVI was less consistent, with regional variation of increased, decreased, or no change to age-standardised prevalence between 1990 and 2020 for all modelled causes (appendix p 8). All age estimates and crude prevalence by sex are shown in the appendix (pp 3–5).

For myopic macular degeneration, we only produced estimates in China because of data sparsity. In 2020, the all-age crude prevalence of vision loss due to myopic macular degeneration was 0·02% (95% UI 0·01–0·03) for blindness and 0·21% (0·14–0·31) for MSVI. The number of people blind due to myopic macular degeneration increased by approximately 200% from 0·103 million (95% UI 0·0678–0·154) people in 1990 to 0·310 million (0·203–0·472) people in 2020. The number of people with MSVI due to myopic macular degeneration increased by approximately 340% from 0·659 million (0·433–1·01) people in 1990 to 2·93 million (1·96–4·38) people in 2020. In 2020, blindness due to myopic macular degeneration was more prevalent than blindness due to diabetic retinopathy (0·232 million people [0·156–0·348]), and similar to age-related macular degeneration (0·327 million people [0·226–0·447], while MSVI due to myopic macular degeneration was more prevalent than diabetic retinopathy (0·882 million people [0·632–1·18]), glaucoma (0·889 million people [0·700–1·11]), and age-related macular degeneration (2·36 million people [1·88–2·89]).

## Discussion

Our results show that both cataract and undercorrected refractive error are among the three leading causes of blindness and MSVI in 2020. We estimate that worldwide there are over 15 million adults aged 50 years and older who are blind due to cataract, and more than 86 million who have MSVI due to undercorrected refractive error. In 2020, blindness due to cataract and undercorrected refractive error composes 50% of all global blindness, and MSVI due to cataract and undercorrected refractive error composes 75% of all global MSVI. The burden of total blindness due to cataract and undercorrected refractive error has notable regional variability, but it has not been fully addressed in any world region, including high-income regions. The other main causes—glaucoma, age-related macular degeneration, and diabetic retinopathy—collectively contribute to over 6 million blind adults aged 50 years and older and over 13 million adults aged 50 years and older with MSVI in 2020. With the exception of diabetic retinopathy, age-standardised prevalence due to all of the causes of blindness we report here has decreased between 1990 and 2020. Given that the vast majority of vision impairment and blindness caused by cataract, undercorrected refractive error, diabetic retinopathy, and glaucoma can be avoided with early detection and timely intervention, a large potential in reducing morbidity for these causes remains.

The WHA defined cataracts and refractive error as avoidable causes of vision impairment because they are so effectively treated with surgery and spectacles, respectively. The WHA GAP set a target of a 25% reduction in crude prevalence of avoidable vision impairment from 2010 to 2019. It specifically highlighted the importance of reducing avoidable vision impairment prevalence in those aged 50 years and older given the majority of vision impairment occurs at older ages.^8^ We estimated that there has been an increase in all-age prevalence of avoidable vision impairment and no change in prevalence for adults aged 50 years and older during this time period, demonstrating that the WHA GAP goal was not met. This lack of change was driven primarily by almost no change in crude and age-standardised prevalence of avoidable MSVI, and a lower-than-necessary decrease in the age-standardised prevalence of avoidable blindness. Cases of avoidable blindness and MSVI both increased in people aged 50 years and older. We therefore note that the GAP target was not met and that ageing populations and the strong association between vision impairment and age were an important barrier in reaching the target. With increasing need for prevention and treatment, eye care services have not managed to sufficiently keep up with demand to meet the target. However, the fact that the change in crude prevalence was better for avoidable blindness (–14·4%) than for avoidable MSVI (1·6%) suggests that the resources put into reducing blindness and visual impairment have been appropriately targeted at reducing blindness ahead of MSVI.

In 2020, cataract remained the first or second leading cause of blindness and MSVI in all world regions.^1^ Cataract can at present only be treated operatively, by a trained surgeon within a system that has capacity to deliver surgeries and manage any postoperative complications. Alleviation efforts have taken the form of mass campaigns, especially in remote areas, and increasing the capacity for and accessibility of surgical services. Because prevalence increases with age, and is higher in women than in men, it remains an important focus for vision loss alleviation and for addressing gender equity. Outreach screening has been shown to enhance equity of access among underserved groups such as women and the elderly.^19^ We therefore recommend developing robust eye care systems supplemented by community outreach as principal strategies.

Undercorrected refractive error is an important cause of vision impairment and is easily treatable by spectacles, contact lenses, or refractive surgery. Myopia, hyperopia, and astigmatism are the main causes of distance vision impairment. Uncorrected aphakia has greatly declined since 1990 because of the use of intraocular lenses during cataract surgery.^20^ Conversely, the prevalence of myopia has been rapidly increasing, especially in urban areas.^21^ The development of refractive services, including the provision of affordable spectacles are the main strategies to address uncorrected refractive errors.

Although cataract and undercorrected refractive error accounted for the majority of blindness and MSVI, glaucoma, age-related macular degeneration, and diabetic retinopathy also caused moderate or worse vision impairment in almost 20 million people aged 50 years and older in 2020. These diseases are not easily treatable.

Glaucoma was ranked as the second leading cause of blindness and fourth leading cause of MSVI, and therefore the most common cause of irreversible blindness, and the second most common cause of irreversible MSVI. Glaucoma in eyes with dense cataract might not be detected, and therefore glaucoma-related irreversible blindness was probably undercounted. With respect to the prevention of glaucoma-related vision impairment, tonometry has not proven to be a useful screening technique and visual acuity assessment is not useful because vision impairment is a late symptom of glaucomatous optic neuropathy.^22^ Once detected, therapy for glaucoma can arrest or slow its deterioration in the majority of cases,^23^ hence the importance of improving systems of surveillance, highlighting risk among family members of cases, and effectiveness of care once treatment is initiated.

The age-standardised prevalence of blindness due to age-related macular degeneration declined by almost 30% from 1990 to 2020. This decrease was probably associated with the widespread clinical introduction of anti-VEGF therapy for exudative age-related macular degeneration.^24^ Since the majority of patients with age-related macular degeneration show the currently untreatable non-exudative form that can progress to atrophy of the foveal centre (geographic atrophy), the development of treatments and prophylactic measures specifically against non-exudative age-related macular degeneration are warranted.

Although diabetic retinopathy was the smallest contributor to blindness in 2020 compared with undercorrected refractive error, cataract, age-related macular degeneration, and glaucoma, it was the only cause of blindness that showed a global increase in age-standardised prevalence between 1990 and 2020. With a projected more than 600 million people living with diabetes by 2040,^25^ and because people with diabetes live increasingly longer, the number of people with diabetic retinopathy and resulting vision impairment is expected to rapidly rise.^26^, ^27^ Our study shows that diabetic retinopathy continues to be an identifiable cause of vision impairment. This is of particular concern in younger, economically active age groups. Our study also shows regional differences in temporal change of blindness due to diabetic retinopathy, with increases in many regions of Asia and sub-Saharan Africa, as well as high-income North America. Of concern is that, in comparison with cataract and undercorrected refractive error, the management of severe diabetic retinopathy requires a disproportionate amount of resources, including availability of ophthalmologists who are trained in laser and surgery.^28^, ^29^

The strengths of this updated review and analysis for 2020 include the substantial addition of new data sources disaggregated by cause and the wider geographical region that these sources cover. In particular, the addition of new RAAB surveys improved geographic coverage, and the age disaggregation of many of these surveys improved the fitting of age patterns to the data. However, there remain several regions such as central sub-Saharan Africa, central and eastern Europe, and central Asia, with little or no population-based data where estimates rely on extrapolation from other regions. There are surprisingly few data from high-income regions—only 19 studies included here reported cause-specific vision impairment in a high-income location, and all but three of these took place more than a decade ago. Sparse data also limited the certainty of estimates of temporal trends and age patterns, particularly in children and young adults for undercorrected refractive error. Minimum ages set for inclusion of data by different causes was agreed with members of the VLEG but this consensus approach could be considered a limitation. A considerable proportion of the blindness burden was listed as “residual causes”. This “other” category is the result of a number of factors such as surveys not committing to a cause for each reported case of vision loss, the truncated list of causes given in published materials, and causes for which we have very limited data such as onchocerciasis, trachoma, xerophthalmia, ocular trauma, corneal diseases, amblyopia, and retinopathy of prematurity. With trachoma, for example, WHO reports that the numbers at risk have dropped from 1·5 billion in 2002 to 142 million in 2019 and the number with trichiasis from 7·6 million to 2·5 million.^30^ RAAB studies were adjusted to reference non-RAAB studies using adjustments generated for all-causes of MSVI and blindness impairment combined because these models were more data rich. However, these adjustments might not be the same for conditions affecting the fundus. Regional-level data might be misleading by masking the diversity of existing situations within countries and even within communities.^31^, ^32^ Greater sociodemographic granularity in data collection is needed to identify populations at risk and capture actual progress in the provision of equitable eye care services.

This report highlights a need for more data on the causes of vision impairment, where we found data sources to be sparse, particularly in children and young adults, high-income locations, and in sub-Saharan Africa. The development of RAABs has led to increasing coverage of low-income and middle-income countries, but not for younger age groups. Also, up-to-date, population-representative data from high-income settings has lagged behind. Better age and geographical data coverage would allow for more detailed analyses of individual country differences and a breakdown of which diseases most impact vision in children.

This report represents a major update of global and regional data on the causes of prevalence of blindness and vision impairment and adds to our understanding of temporal change over 30 years. Over the past decade the prevalence of avoidable visual impairment has decreased in adults aged 50 years and older, but it has not reached the targeted 25% reduction delineated in the WHA GAP. The principal reason from a global perspective is the failure of eye care services to keep pace with the ageing and growth of populations, yet the reduction in age-standardised prevalence of blindness due to causes such as cataract, glaucoma, age-related macular degeneration, and undercorrected refractive error was a reassuring change in the right direction. The report demonstrates the considerable inter-regional differences that exist and highlights areas which require particular attention, such as blindness due to diabetic retinopathy, which was the only cause to increase in age-standardised blindness prevalence over three decades.

Correspondence to: Prof Rupert R A Bourne, Vision and Eye Research Institute, Anglia Ruskin University, Cambridge CB1 1PT, UK rb@rupertbourne.co.uk

## Data sharing

More than ten authors have accessed and verified the data. To download the data used in these analyses, please visit the Global Health Data Exchange GBD 2019 website.
