# Correction to *Lancet Glob Health* 2021; 2: 144–60

**DOI:** 10.1016/S2214-109X(21)00050-4

**Published:** 2021-02-10

*GBD 2019 Blindness and Vision Impairment Collaborators. Causes of blindness and vision impairment in 2020 and trends over 30 years, and prevalence of avoidable blindness in relation to VISION 2020: the Right to Sight: an analysis for the Global Burden of Disease Study.* Lancet Glob Health *2021;* 2: *144–60*—In this paper, the name of one of the GBD 2019 Blindness and Vision Impairment Collaborators should have been Jaimie D Steinmetz. This correction has been made to the online version as of Feb 10, 2021.

