# Supplementary material for: Causes of blindness and vision impairment in 2020 and trends over 30 years, and prevalence of avoidable blindness in relation to VISION 2020: the Right to Sight: an analysis for the Global Burden of Disease Study
Source: Lancet Glob Health. 2020 Dec 1;9(2):e144–60. doi: 10.1016/S2214-109X(20)30489-7 (PMC7820391; doi:10.1016/S2214-109X(20)30489-7)
Supplement: Supplementary appendix [file mmc1.pdf]

THE LANCET
Global Health

Supplementary appendix

This appendix formed part of the original submission and has been peer reviewed. We post it as supplied by the authors.

Supplement to: GBD 2019 Blindness and Vision Impairment Collaborators on behalf of the Vision Loss Expert Group of the Global Burden of Disease Study. Causes of blindness and vision impairment in 2020 and trends over 30 years, and prevalence of avoidable blindness in relation to VISION 2020: the Right to Sight: an analysis for the Global Burden of Disease Study. *Lancet Glob Health* 2020; published online Dec 1. http://dx.doi.org/10.1016/S2214-109X(20)30489-7.

APPENDIX: SUPPLEMENTARY RESULTS

Contents

Table 1A. Cases and age-standardized prevalence in 2020 for blindness by cause, and percent change from 1990 to 2020 (all ages)

Table 1B. Cases and age-standardized prevalence in 2020 for moderate and severe vision impairment by cause, and percent change from 1990 to 2020 (all ages)

Table 2A. Aged 50+ crude prevalence (per 1000 people) by cause and severity in 2020.

Table 2B. All age crude prevalence (per 1000 people) by cause and severity in 2020.

Table 3. Change in all-age, crude prevalence in 2010-2019 to assess the success of the World Health Assembly's global action plan to reduce avoidable vision impairment.

Figure 1. Coverage of data sources (1980-2018) with cause-specific vision impairment prevalence data for cataract, age-related macular degeneration, glaucoma and diabetic retinopathy (coverage is presented as numbers of sources per region).

Figure 2. Change in regional age-standardized prevalence of moderate and severe vision impairment between 1990 and 2020 by cause

Contributors

Table 1A. Cases and age-standardized prevalence in 2020 for blindness by cause, and percent change from 1990 to 2020 (all ages)

	Cataract				Undercorrected refractive error			
**Super Region**	**Cases in 1000s (95% UI)**	**% change 1990 to 2020 (95% UI)**	**Age-standardized rate per 1,000 (95% UI)**	**% change 1990 to 2020 (95% UI)**	**Cases in 1000s (95% UI)**	**% change 1990 to 2020 (95% UI)**	**Age-standardized rate per 1,000 (95% UI)**	**% change 1990 to 2020 (95% UI)**
Central Europe, Eastern Europe, and Central Asia	291 (234–357)	-3·9 (-6·6—1·2)	0·463 (0·372–0·564)	-34·3 (-36·0—32·5)	29·4 (23·3–36·0)	4·0 (1·0–7·1)	0·0586 (0·0459–0·0728)	-8·0 (-10·8—5·0)
High-income	506 (411–620)	44·6 (36·4–53·2)	0·234 (0·189–0·283)	-23·3 (-25·0—21·7)	80·0 (63·6–97·6)	7·4 (1·9–13·0)	0·0591 (0·0454–0·0749)	-21·0 (-23·4—18·7)
Latin America and Caribbean	1100 (911–1320)	70·0 (64·5–75·7)	1·86 (1·54–2·23)	-43·9 (-44·9—42·8)	218 (180–258)	57·4 (49·8–64·6)	0·353 (0·291–0·416)	-28·9 (-30·8—27·1)
North Africa and Middle East	844 (677–1040)	28·3 (23·7–33·3)	2·11 (1·69–2·61)	-53·8 (-55·4—52·3)	190 (156–229)	49·3 (38·8–58·2)	0·348 (0·286–0·413)	-32·4 (-35·3—29·7)
South Asia	6350 (5370–7420)	43·1 (37·1–49·3)	5·12 (4·36–5·94)	-47·3 (-48·9—45·5)	1520 (1260–1770)	23·8 (18·4–29·0)	1·00 (0·825–1·17)	-48·9 (-50·8—46·7)
Southeast Asia, East Asia, and Oceania	6310 (5320–7450)	54·5 (48·7–60·2)	2·42 (2·05–2·83)	-43·4 (-45·0—41·4)	1410 (1160–1650)	52·7 (43·4–62·2)	0·534 (0·443–0·622)	-23·0 (-26·3—19·9)
Sub-Saharan Africa	1600 (1360–1860)	57·2 (54·0–60·8)	3·68 (3·10–4·33)	-30·9 (-32·1—29·7)	254 (212–303)	103·2 (96·5–110·6)	0·380 (0·310–0·446)	-13·3 (-15·4—11·1)
Global	17000 (14400–19900)	47·9 (43·7–52·0)	2·05 (1·74–2·40)	-32·7 (-34·2—31·2)	3700 (3100–4290)	39·9 (35·0–44·7)	0·444 (0·374–0·514)	-25·2 (-27·4—23·1)
**Super Region**	**Glaucoma**				**Age-related macular degeneration**			
Central Europe, Eastern Europe, and Central Asia	178 (139–220)	5·5 (2·0–9·3)	0·273 (0·213–0·335)	-31·4 (-32·8—30·0)	62·8 (43·5–84·4)	21·8 (17·2–27·4)	0·0961 (0·0672–0·129)	-16·8 (-18·5—14·9)
High-income	786 (623–965)	60·2 (52·9–69·0)	0·310 (0·246–0·381)	-23·2 (-24·5—21·9)	596 (456–770)	48·1 (40·7–56·2)	0·238 (0·182–0·305)	-28·6 (-29·9—27·2)
Latin America and Caribbean	335 (257–412)	105·4 (98·3–113·6)	0·576 (0·440–0·707)	-36·2 (-37·6—34·8)	71·5 (49·4–97·4)	141·5 (131·0–152·1)	0·121 (0·0835–0·164)	-21·2 (-23·4—18·8)
North Africa and Middle East	463 (355–579)	64·8 (57·6–72·3)	1·25 (0·957–1·55)	-40·9 (-43·0—39·0)	195 (137–264)	105·2 (95·0–115·8)	0·488 (0·340–0·656)	-23·3 (-26·6—20·1)
South Asia	579 (441–729)	75·3 (64·7–86·1)	0·495 (0·375–0·619)	-38·7 (-41·0—36·1)	298 (201–424)	52·7 (41·6–65·0)	0·231 (0·158–0·323)	-41·2 (-44·2—37·7)
Southeast Asia, East Asia, and Oceania	757 (577–961)	52·5 (44·5–61·3)	0·294 (0·225–0·367)	-48·5 (-51·3—45·5)	495 (342–680)	103·9 (91·8–116·4)	0·184 (0·128–0·251)	-27·3 (-31·2—22·8)
Sub-Saharan Africa	511 (400–629)	70·2 (65·2–75·2)	1·45 (1·14–1·77)	-23·5 (-25·4—21·5)	131 (92·2–179)	78·6 (70·3–86·7)	0·329 (0·231–0·448)	-19·2 (-23·0—15·8)
Global	3610 (2810–4420)	61·8 (57·2–66·7)	0·447 (0·348–0·546)	-31·9 (-33·0—30·6)	1850 (1350–2430)	69·6 (64·2–75·2)	0·227 (0·166–0·298)	-28·0 (-30·0—25·6)
**Super Region**	**Diabetic retinopathy**				**Residual causes of vision loss**			
Central Europe, Eastern Europe, and Central Asia	13·7 (9·44–19·7)	24·0 (15·5–32·9)	0·0222 (0·0154–0·0319)	-7·1 (-12·6—1·1)	842 (725–955)	7·4 (5·2–9·9)	1·49 (1·30–1·67)	-18·1 (-19·8—16·4)
High-income	161 (116–227)	43·3 (34·7–52·1)	0·0859 (0·0618–0·120)	-13·8 (-17·9—9·7)	879 (759–1000)	16·4 (12·1–21·0)	0·608 (0·520–0·699)	-18·3 (-20·1—16·5)
Latin America and Caribbean	254 (186–348)	108·0 (98·2–118·0)	0·408 (0·298–0·559)	-20·4 (-22·9—17·9)	1680 (1450–1910)	78·0 (70·2–85·2)	2·77 (2·39–3·15)	-24·7 (-26·3—23·1)
North Africa and Middle East	73·3 (50·3–106)	166·4 (147·0–188·8)	0·154 (0·106–0·226)	-0·5 (-7·5–7·4)	1330 (1140–1540)	59·4 (53·7–65·9)	2·65 (2·26–3·09)	-33·3 (-36·1—30·7)
South Asia	197 (136–285)	146·0 (122·0–173·5)	0·131 (0·0902–0·187)	6·8 (-2·5–17·1)	3000 (2570–3450)	23·8 (15·5–32·0)	1·98 (1·70–2·28)	-47·9 (-52·7—43·0)
Southeast Asia, East Asia, and Oceania	325 (222–478)	247·8 (212·2–279·1)	0·113 (0·0777–0·164)	43·2 (31·9–53·9)	5790 (5010–6530)	61·9 (53·6–70·2)	2·29 (2·01–2·57)	-18·6 (-21·2—16·0)
Sub-Saharan Africa	49·7 (35·3–71·3)	174·8 (160·2–190·3)	0·0936 (0·0659–0·131)	21·0 (15·0–27·5)	2530 (2210–2860)	70·4 (65·5–75·1)	3·98 (3·40–4·59)	-27·8 (-29·9—25·9)
Global	1070 (763–1510)	130·7 (120·8–140·4)	0·126 (0·0894–0·177)	10·5 (6·6–14·0)	16000 (14000–18100)	48·6 (44·4–53·1)	1·95 (1·71–2·19)	-20·5 (-23·1—18·2)

Table 1B. Cases and age-standardized prevalence in 2020 for moderate and severe vision impairment by cause, and percent change from 1990 to 2020 (all ages)

	Cataract				Undercorrected refractive error			
**Super Region**	**Cases in 1000s (95% UI)**	**% change 1990 to 2020 (95% UI)**	**Age-standardized rate per 1,000 (95% UI)**	**% change 1990 to 2020 (95% UI)**	**Cases in 1000s (95% UI)**	**% change 1990 to 2020 (95% UI)**	**Age-standardized rate per 1,000 (95% UI)**	**% change 1990 to 2020 (95% UI)**
Central Europe, Eastern Europe, and Central Asia	3170 (2620–3730)	49·5 (46·2–52·7)	4·91 (4·08–5·77)	1·1 (-0·4–2·6)	9660 (8560–10900)	13·4 (11·0–15·5)	18·5 (16·5–20·6)	-3·0 (-3·7—2·2)
High-income	8210 (6960–9570)	95·3 (89·4–101·1)	3·47 (2·96–4·03)	-1·2 (-2·2—0·1)	17100 (15200–18900)	27·1 (23·4–30·9)	13·7 (12·1–15·3)	-2·1 (-2·8—1·5)
Latin America and Caribbean	4660 (3940–5390)	200·4 (194·4–207·1)	7·89 (6·67–9·15)	-0·9 (-2·1–0·3)	14700 (13000–16300)	72·0 (65·5–78·0)	23·9 (21·2–26·5)	-6·1 (-6·9—5·5)
North Africa and Middle East	5400 (4570–6300)	181·0 (173·1–189·0)	13·3 (11·3–15·5)	0·6 (-2·0–3·5)	12800 (11400–14400)	90·7 (84·6–96·7)	22·4 (20·0–25·0)	-7·3 (-8·8—5·8)
South Asia	28700 (24700–33500)	174·2 (166·3–182·7)	21·5 (18·5–24·9)	1·1 (-0·8–3·0)	53900 (47800–60900)	71·3 (67·8–75·0)	33·7 (29·9–38·1)	-21·7 (-22·7—20·6)
Southeast Asia, East Asia, and Oceania	28200 (24400–32400)	229·3 (223·0–235·5)	10·8 (9·34–12·4)	14·1 (12·3–15·9)	39700 (35400–44600)	79·0 (70·9–86·4)	16·0 (14·3–17·8)	-0·5 (-2·2–1·2)
Sub-Saharan Africa	5050 (4340–5780)	155·7 (150·8–161·0)	12·0 (10·3–13·7)	11·7 (10·0–13·5)	9620 (8480–10900)	134·7 (131·0–138·3)	12·4 (11·0–13·8)	2·3 (1·0–3·5)
Global	83500 (71800–96000)	170·7 (166·7–174·7)	10·1 (8·65–11·5)	18·9 (17·7–20·3)	157000 (140000–176000)	65·8 (61·7–69·6)	19·1 (17·1–21·3)	-5·4 (-6·1—4·7)
**Super Region**	**Glaucoma**				**Age-related macular degeneration**			
Central Europe, Eastern Europe, and Central Asia	213 (167–270)	43·7 (40·0–47·6)	0·323 (0·253–0·408)	-2·9 (-4·6—1·3)	228 (182–283)	51·1 (47·8–54·3)	0·345 (0·275–0·427)	4·8 (3·1–6·5)
High-income	596 (468–763)	103·0 (95·6–111·4)	0·239 (0·187–0·305)	-0·2 (-1·5–1·0)	739 (585–918)	76·8 (70·5–83·3)	0·306 (0·243–0·376)	-9·4 (-11·2—7·5)
Latin America and Caribbean	499 (391–623)	191·6 (185·1–199·1)	0·847 (0·663–1·06)	-4·7 (-6·1—3·1)	334 (270–408)	202·1 (192·9–212·0)	0·559 (0·454–0·685)	1·6 (-1·2–4·5)
North Africa and Middle East	326 (252–420)	148·2 (139·2–157·8)	0·823 (0·629–1·06)	-9·7 (-12·8—6·5)	494 (391–614)	166·1 (156·6–175·6)	1·20 (0·955–1·48)	-0·5 (-4·0–2·9)
South Asia	955 (747–1200)	144·9 (136·1–154·3)	0·741 (0·584–0·923)	-12·9 (-15·0—10·6)	1220 (972–1510)	121·3 (112·7–130·1)	0·917 (0·736–1·12)	-20·5 (-23·0—18·0)
Southeast Asia, East Asia, and Oceania	1160 (917–1450)	251·4 (241·9–261·9)	0·440 (0·347–0·552)	21·4 (18·3–24·8)	2760 (2210–3380)	214·6 (204·7–224·4)	1·01 (0·817–1·23)	13·8 (10·7–17·1)
Sub-Saharan Africa	392 (308–494)	112·0 (107·2–117·0)	1·00 (0·792–1·25)	-4·3 (-6·2—2·2)	454 (360–566)	132·3 (124·8–140·5)	1·09 (0·871–1·34)	5·6 (2·4–9·0)
Global	4140 (3240–5180)	151·1 (147·1–155·2)	0·502 (0·395–0·627)	8·3 (6·8–9·8)	6230 (5040–7590)	150·2 (145·8–154·7)	0·744 (0·602–0·903)	10·6 (8·7–12·6)
**Super Region**	**Diabetic retinopathy**				**Residual causes of vision loss**			
Central Europe, Eastern Europe, and Central Asia	144 (102–194)	21·8 (17·6–25·9)	0·226 (0·161–0·302)	-10·6 (-13·2—8·0)	4570 (3930–5330)	31·7 (29·1–34·3)	7·38 (6·41–8·54)	-3·8 (-5·1—2·6)
High-income	420 (309–558)	62·3 (56·9–67·8)	0·204 (0·153–0·273)	-7·0 (-9·0—5·2)	4010 (3460–4640)	56·0 (51·1–61·0)	2·44 (2·13–2·79)	0·1 (-1·8–2·2)
Latin America and Caribbean	451 (334–599)	173·1 (164·5–182·1)	0·735 (0·544–0·974)	-1·2 (-3·3–1·0)	3850 (3340–4430)	119·2 (110·9–127·0)	6·41 (5·56–7·36)	-4·3 (-5·7—2·9)
North Africa and Middle East	462 (339–609)	116·9 (107·6–126·1)	0·997 (0·732–1·33)	-19·8 (-23·3—16·3)	2320 (2020–2670)	87·2 (79·2–95·4)	4·37 (3·77–5·02)	-14·9 (-18·7—11·2)
South Asia	445 (324–598)	116·9 (107·8–126·8)	0·309 (0·227–0·409)	-16·0 (-19·2—12·7)	11000 (9650–12500)	90·1 (82·9–97·9)	7·17 (6·31–8·19)	-7·3 (-9·5—4·9)
Southeast Asia, East Asia, and Oceania	1190 (863–1590)	177·3 (162·2–193·0)	0·422 (0·308–0·563)	4·0 (-1·8–9·7)	9970 (8740–11400)	106·6 (97·8–114·7)	3·91 (3·47–4·41)	-1·1 (-3·8–1·7)
Sub-Saharan Africa	165 (121–217)	148·7 (142–156)	0·348 (0·255–0·459)	9·2 (6·5–12·2)	4760 (4170–5390)	67·1 (60·8–73·3)	7·42 (6·46–8·48)	-30·2 (-32·8—27·4)
Global	3280 (2410–4340)	125·0 (119·5–131·0)	0·386 (0·284–0·510)	3·3 (0·7–5·7)	40500 (35500–46000)	79·9 (76·0–83·8)	4·93 (4·36–5·59)	-4·9 (-6·3—3·3)

Table 2A. Aged 50+ crude prevalence (per 1000 people) by cause and severity in 2020.

	Cataract, MSVI			Cataract, blindness			Undercorrected refractive error, MSVI			Undercorrected refractive error, blindness		
Super Region	Male crude prev (95% CI)	Female crude prev (95% CI)	Both crude prev (95% CI)	Male crude prev (95% CI)	Female crude prev (95% CI)	Both crude prev (95% CI)	Male crude prev (95% CI)	Female crude prev (95% CI)	Both crude prev (95% CI)	Male crude prev (95% CI)	Female crude prev (95% CI)	Both crude prev (95% CI)
Central Europe, Eastern Europe, and Central Asia	15·9 (12·9–19·0)	26·5 (21·8–31·6)	22·1 (18·1–26·2)	1·53 (1·22–1·93)	2·21 (1·78–2·76)	1·93 (1·53–2·41)	39·1 (33·1–46·7)	50·9 (43·4–59·8)	45·9 (39·2–54·2)	0·122 (0·0909–0·153)	0·122 (0·0919–0·155)	0·122 (0·0924–0·153)
High-income	14·2 (11·9–16·7)	22·5 (19·0–26·3)	18·6 (15·7–21·8)	0·990 (0·793–1·22)	1·15 (0·926–1·44)	1·08 (0·866–1·34)	18·8 (16·2–21·7)	23·1 (19·9–26·8)	21·1 (18·1–24·4)	0·107 (0·0817–0·132)	0·111 (0·0857–0·137)	0·109 (0·0842–0·134)
Latin America and Caribbean	30·2 (25·2–35·3)	33·2 (27·9–38·9)	31·8 (26·7–37·2)	6·83 (5·57–8·34)	7·81 (6·41–9·45)	7·36 (6·04–8·88)	41·1 (35·2–48·3)	43·2 (37·1–50·6)	42·2 (36·2–49·6)	0·847 (0·650–1·03)	0·990 (0·771–1·20)	0·923 (0·713–1·12)
North Africa and Middle East	41·6 (34·9–49·4)	54·1 (45·5–63·6)	47·7 (40·2–56·3)	5·16 (4·01–6·55)	9·26 (7·31–11·5)	7·18 (5·63–8·94)	42·5 (35·9–50·1)	46·5 (39·3–54·9)	44·5 (37·6–52·7)	0·733 (0·552–0·902)	0·868 (0·660–1·07)	0·799 (0·606–0·982)
Southeast Asia, East Asia, and Oceania	33·1 (28·2–38·4)	47·3 (40·6–54·6)	40·4 (34·6–46·5)	6·10 (5·04–7·32)	10·5 (8·75–12·4)	8·35 (6·96–9·92)	34·5 (29·6–40·5)	40·8 (35·1–47·5)	37·7 (32·4–44·1)	1·29 (1·01–1·58)	1·52 (1·19–1·85)	1·41 (1·10–1·72)
South Asia	75·4 (64·0–88·6)	93·8 (80·2–109)	84·7 (72·2–99·1)	15·7 (13·2–18·4)	21·1 (17·8–24·9)	18·4 (15·5–21·7)	97·8 (83·4–115)	102 (87·4–121)	100 (85·4–118)	2·88 (2·25–3·50)	3·19 (2·48–3·91)	3·04 (2·37–3·70)
Sub-Saharan Africa	35·8 (30·4–41·8)	45·2 (38·6–52·5)	40·8 (34·8–47·4)	9·71 (8·10–11·8)	12·8 (10·7–15·4)	11·4 (9·47–13·7)	28·8 (24·6–34·1)	30·1 (25·6–35·6)	29·5 (25·1–34·9)	1·02 (0·783–1·27)	1·01 (0·770–1·25)	1·02 (0·777–1·25)
Global	35·8 (30·4–41·6)	46·8 (40·0–54·1)	41·5 (35·4–48·2)	6·57 (5·49–7·84)	9·29 (7·79–11·0)	7·99 (6·69–9·47)	43·1 (37–50·5)	47·5 (40·9–55·6)	45·4 (39·1–53·2)	1·16 (0·901–1·40)	1·26 (0·983–1·54)	1·21 (0·944–1·48)

Super Region	Glaucoma, MSVI			Glaucoma, blindness			Age-related macular degeneration, MSVI			Age-related macular degeneration, blindness		
Central Europe, Eastern Europe, and Central Asia	1·34 (1·04–1·70)	1·69 (1·33–2·15)	1·54 (1·21–1·95)	1·35 (1·06–1·66)	1·25 (0·966–1·53)	1·29 (1·00–1·59)	1·31 (1·03–1·62)	1·90 (1·52–2·35)	1·65 (1·32–2·05)	0·354 (0·245–0·480)	0·525 (0·365–0·705)	0·453 (0·313–0·609)
High-income	1·25 (0·981–1·61)	1·54 (1·20–1·98)	1·41 (1·10–1·80)	1·90 (1·52–2·30)	1·81 (1·41–2·26)	1·85 (1·47–2·27)	1·48 (1·17–1·83)	1·97 (1·55–2·45)	1·74 (1·38–2·16)	0·848 (0·627–1·12)	1·89 (1·45–2·40)	1·40 (1·07–1·81)
Latin America and Caribbean	3·87 (3·05–4·84)	3·44 (2·69–4·33)	3·64 (2·85–4·55)	2·69 (2·08–3·32)	2·22 (1·68–2·74)	2·44 (1·87–3·00)	2·16 (1·73–2·65)	2·67 (2·17–3·27)	2·43 (1·97–2·97)	0·367 (0·247–0·508)	0·653 (0·453–0·891)	0·520 (0·359–0·710)
North Africa and Middle East	3·14 (2·43–4·04)	3·04 (2·33–3·91)	3·09 (2·38–3·98)	4·97 (3·83–6·20)	3·79 (2·86–4·79)	4·39 (3·36–5·49)	4·38 (3·46–5·51)	5·00 (3·95–6·19)	4·68 (3·71–5·83)	1·45 (0·997–1·95)	2·26 (1·57–3·07)	1·85 (1·29–2·51)
Southeast Asia, East Asia, and Oceania	1·72 (1·35–2·14)	1·77 (1·39–2·24)	1·75 (1·38–2·18)	1·19 (0·913–1·52)	1·08 (0·820–1·37)	1·13 (0·866–1·44)	3·81 (3·04–4·65)	4·47 (3·58–5·45)	4·15 (3·33–5·08)	0·572 (0·392–0·791)	0·901 (0·622–1·24)	0·741 (0·512–1·01)
South Asia	3·22 (2·51–4·07)	2·71 (2·11–3·41)	2·96 (2·32–3·72)	2·12 (1·63–2·66)	1·47 (1·12–1·87)	1·79 (1·37–2·26)	3·58 (2·84–4·42)	4·00 (3·19–4·95)	3·79 (3·01–4·69)	0·886 (0·594–1·25)	0·954 (0·643–1·36)	0·920 (0·620–1·31)
Sub-Saharan Africa	3·73 (2·93–4·71)	3·47 (2·73–4·35)	3·59 (2·82–4·53)	5·31 (4·19–6·52)	4·13 (3·21–5·12)	4·69 (3·66–5·77)	3·94 (3·12–4·93)	4·36 (3·45–5·45)	4·16 (3·3–5·19)	0·891 (0·624–1·22)	1·47 (1·03–2·00)	1·20 (0·840–1·64)
Global	2·21 (1·73–2·77)	2·15 (1·69–2·69)	2·18 (1·70–2·72)	2·08 (1·64–2·55)	1·73 (1·33–2·12)	1·90 (1·48–2·32)	3·03 (2·43–3·72)	3·51 (2·84–4·26)	3·28 (2·65–3·99)	0·729 (0·518–0·982)	1·19 (0·878–1·55)	0·970 (0·706–1·28)

Super Region	Diabetic retinopathy, MSVI			Diabetic retinopathy, blindness			Residual causes of vision loss, MSVI			Residual causes of vision loss, blindness		
Central Europe, Eastern Europe, and Central Asia	0·795 (0·553–1·09)	1·10 (0·778–1·49)	0·974 (0·683–1·32)	0·0256 (0·0157–0·0397)	0·130 (0·0875–0·188)	0·086 (0·0577–0·125)	23·5 (19·7–28·1)	35·2 (29·9–41·5)	30·3 (25·6–35·9)	4·19 (3·50–4·97)	5·05 (4·23–5·92)	4·69 (3·95–5·46)
High-income	0·765 (0·549–1·03)	1·04 (0·742–1·41)	0·910 (0·654–1·23)	0·195 (0·130–0·289)	0·445 (0·315–0·615)	0·328 (0·229–0·463)	6·1 (5·12–7·27)	8·21 (6·84–9·74)	7·22 (6·06–8·56)	1·35 (1·13–1·59)	1·26 (1·05–1·49)	1·30 (1·09–1·53)
Latin America and Caribbean	2·91 (2·10–3·93)	2·89 (2·10–3·88)	2·90 (2·12–3·90)	1·56 (1·07–2·20)	1·42 (1·00–1·95)	1·48 (1·04–2·07)	19·1 (16–22·8)	20·4 (17·0–24·3)	19·8 (16·5–23·6)	8·40 (6·88–10·0)	8·27 (6·81–9·86)	8·33 (6·88–9·89)
North Africa and Middle East	3·36 (2·43–4·57)	4·24 (3·06–5·71)	3·79 (2·74–5·13)	0·621 (0·405–0·931)	0·536 (0·356–0·813)	0·579 (0·381–0·872)	9·9 (8·02–12·1)	10·8 (8·82–13·1)	10·3 (8·46–12·6)	7·38 (5·93–8·96)	7·59 (6·06–9·30)	7·48 (6·00–9·11)
Southeast Asia, East Asia, and Oceania	1·52 (1·09–2·06)	1·79 (1·29–2·42)	1·66 (1·20–2·25)	0·361 (0·237–0·536)	0·422 (0·276–0·627)	0·392 (0·257–0·583)	10·3 (8·81–11·9)	13·3 (11·5–15·4)	11·8 (10·2–13·7)	5·46 (4·57–6·44)	6·12 (5·14–7·14)	5·80 (4·86–6·78)
South Asia	1·07 (0·765–1·45)	1·35 (0·96–1·83)	1·21 (0·868–1·64)	0·405 (0·269–0·593)	0·538 (0·358–0·801)	0·472 (0·315–0·700)	20·1 (16·8–24)	22·8 (19·1–27·3)	21·5 (18·0–25·7)	5·43 (4·42–6·55)	4·97 (4·03–6·04)	5·20 (4·25–6·25)
Sub-Saharan Africa	1·15 (0·821–1·56)	1·37 (0·984–1·87)	1·27 (0·906–1·71)	0·274 (0·180–0·411)	0·346 (0·237–0·496)	0·312 (0·211–0·452)	22 (18·5–26·2)	22·3 (18·6–26·6)	22·1 (18·5–26·5)	11·2 (9·14–13·5)	10·8 (8·79–12·9)	11·00 (8·94–13·2)
Global	1·42 (1·03–1·91)	1·68 (1·22–2·24)	1·55 (1·13–2·08)	0·405 (0·274–0·582)	0·498 (0·343–0·712)	0·434 (0·312–0·650)	13·2 (11·2–15·5)	16·4 (13·9–19·1)	14·9 (12·7–17·4)	5·12 (4·25–6·03)	5·24 (4·39–6·13)	5·19 (4·32–6·08)

Table 2B. All age crude prevalence (per 1000 people) by cause and severity in 2020.

Super Region	Cataract, MSVI Male crude prev (95% CI)	Cataract, MSVI Female crude prev (95% CI)	Cataract, MSVI Both crude prev (95% CI)	Cataract, blindness Male crude prev (95% CI)	Cataract, blindness Female crude prev (95% CI)	Cataract, blindness Both crude prev (95% CI)	Undercorrected refractive error, MSVI Male crude prev (95% CI)	Undercorrected refractive error, MSVI Female crude prev (95% CI)	Undercorrected refractive error, MSVI Both crude prev (95% CI)	Undercorrected refractive error, blindness Male crude prev (95% CI)	Undercorrected refractive error, blindness Female crude prev (95% CI)	Undercorrected refractive error, blindness Both crude prev (95% CI)
Central Europe, Eastern Europe, and Central Asia	4·91 (4·04–5·81)	10·1 (8·28–11·9)	7·60 (6·28–8·95)	0·513 (0·409–0·627)	0·866 (0·699–1·07)	0·698 (0·561–0·855)	19·2 (17·0–21·7)	26·7 (23·7–30·3)	23·1 (20·5–26·1)	0·0689 (0·0544–0·0856)	0·0718 (0·0568–0·0881)	0·0704 (0·0558–0·0863)
High-income	5·58 (4·72–6·52)	9·45 (8·01–11·0)	7·55 (6·39–8·80)	0·414 (0·334–0·500)	0·514 (0·419–0·636)	0·465 (0·378–0·569)	15·4 (13·6–17·1)	16·0 (14·3–17·7)	15·7 (14·0–17·4)	0·0689 (0·0544–0·0838)	0·0780 (0·0627–0·0946)	0·0736 (0·0584–0·0897)
Latin America and Caribbean	6·97 (5·89–8·09)	8·49 (7·19–9·83)	7·74 (6·54–8·96)	1·64 (1·35–1·96)	2·02 (1·67–2·41)	1·83 (1·51–2·20)	22·2 (19·7–24·7)	26·6 (23·5–29·6)	24·4 (21·7–27·1)	0·325 (0·266–0·386)	0·399 (0·330–0·469)	0·363 (0·299–0·428)
North Africa and Middle East	7·35 (6·22–8·59)	9·83 (8·34–11·4)	8·55 (7·24–9·98)	0·963 (0·762–1·19)	1·74 (1·39–2·13)	1·34 (1·07–1·64)	19·5 (17·3–21·8)	21·2 (18·9–23·8)	20·3 (18·0–22·7)	0·276 (0·225–0·333)	0·328 (0·269–0·393)	0·301 (0·246–0·362)
Southeast Asia, East Asia, and Oceania	10·2 (8·78–11·7)	15·6 (13·5–18·0)	12·9 (11·1–14·8)	2·07 (1·74–2·45)	3·70 (3·14–4·35)	2·88 (2·43–3·40)	16·4 (14·6–18·4)	19·9 (17·7–22·4)	18·1 (16·1–20·3)	0·592 (0·485–0·692)	0·693 (0·569–0·812)	0·642 (0·528–0·752)
South Asia	13·5 (11·6–15·9)	17·8 (15·3–20·6)	15·6 (13·4–18·2)	2·88 (2·44–3·36)	4·05 (3·43–4·73)	3·45 (2·92–4·03)	27·2 (24·1–30·7)	31·4 (27·7–35·4)	29·3 (25·9–33·1)	0·774 (0·645–0·900)	0·876 (0·731–1·03)	0·824 (0·686–0·961)
Sub-Saharan Africa	3·77 (3·24–4·36)	5·27 (4·54–6·01)	4·53 (3·89–5·19)	1·19 (1·01–1·39)	1·68 (1·42–1·94)	1·44 (1·22–1·67)	8·22 (7·22–9·31)	9·02 (7·96–10·2)	8·63 (7·61–9·77)	0·223 (0·186–0·266)	0·233 (0·195–0·279)	0·228 (0·190–0·272)
Global	8·74 (7·50–10·1)	12·4 (10·7–14·3)	10·6 (9·1–12·2)	1·71 (1·45–2·02)	2·60 (2·21–3·04)	2·16 (1·83–2·53)	18·5 (16·5–20·7)	21·4 (19·1–23·9)	20·0 (17·8–22·3)	0·441 (0·373–0·510)	0·497 (0·413–0·581)	0·469 (0·394–0·545)

Super Region	Glaucoma, MSVI			Glaucoma, blindness			Age-related macular degeneration, MSVI			Age-related macular degeneration, blindness		
Central Europe, Eastern Europe, and Central Asia	0·391 (0·303–0·497)	0·621 (0·487–0·789)	0·511 (0·401–0·647)	0·394 (0·309–0·484)	0·458 (0·355–0·563)	0·428 (0·333–0·526)	0·381 (0·302–0·473)	0·699 (0·558–0·865)	0·547 (0·437–0·678)	0·104 (0·0717–0·141)	0·193 (0·134–0·259)	0·150 (0·104–0·202)
High-income	0·466 (0·364–0·596)	0·628 (0·492–0·808)	0·548 (0·431–0·702)	0·703 (0·564–0·853)	0·741 (0·576–0·924)	0·722 (0·573–0·887)	0·550 (0·436–0·680)	0·805 (0·634–1·00)	0·680 (0·538–0·843)	0·315 (0·233–0·416)	0·773 (0·593–0·983)	0·548 (0·419–0·708)
Latin America and Caribbean	0·834 (0·656–1·04)	0·825 (0·644–1·04)	0·829 (0·650–1·04)	0·581 (0·448–0·715)	0·533 (0·404–0·657)	0·556 (0·426–0·684)	0·465 (0·373–0·571)	0·642 (0·521–0·784)	0·555 (0·449–0·678)	0·0795 (0·0535–0·110)	0·157 (0·109–0·213)	0·119 (0·0822–0·162)
North Africa and Middle East	0·516 (0·399–0·662)	0·517 (0·397–0·666)	0·517 (0·398–0·664)	0·816 (0·628–1·02)	0·645 (0·486–0·814)	0·733 (0·562–0·916)	0·718 (0·568–0·904)	0·850 (0·673–1·05)	0·782 (0·619–0·973)	0·238 (0·164–0·322)	0·385 (0·268–0·522)	0·309 (0·217–0·419)
Southeast Asia, East Asia, and Oceania	0·500 (0·395–0·624)	0·561 (0·441–0·709)	0·530 (0·418–0·662)	0·348 (0·267–0·442)	0·342 (0·259–0·435)	0·345 (0·263–0·438)	1·11 (0·885–1·35)	1·41 (1·13–1·72)	1·26 (1·01–1·54)	0·167 (0·115–0·232)	0·286 (0·198–0·392)	0·226 (0·156–0·310)
South Asia	0·547 (0·426–0·690)	0·489 (0·381–0·615)	0·519 (0·406–0·652)	0·360 (0·277–0·453)	0·266 (0·201–0·336)	0·314 (0·240–0·396)	0·607 (0·482–0·749)	0·721 (0·576–0·891)	0·663 (0·528–0·820)	0·151 (0·102–0·212)	0·173 (0·117–0·246)	0·162 (0·109–0·230)
Sub-Saharan Africa	0·350 (0·275–0·441)	0·354 (0·279–0·444)	0·352 (0·276–0·443)	0·496 (0·392–0·609)	0·422 (0·328–0·522)	0·458 (0·359–0·564)	0·369 (0·292–0·460)	0·445 (0·352–0·555)	0·407 (0·323–0·507)	0·0838 (0·0587–0·114)	0·151 (0·106–0·204)	0·118 (0·0827–0·160)
Global	0·506 (0·398–0·634)	0·543 (0·427–0·681)	0·525 (0·411–0·656)	0·478 (0·377–0·585)	0·437 (0·337–0·537)	0·457 (0·356–0·561)	0·694 (0·558–0·853)	0·887 (0·718–1·08)	0·790 (0·639–0·962)	0·168 (0·119–0·226)	0·302 (0·223–0·393)	0·233 (0·171–0·309)

Super Region	Diabetic retinopathy, MSVI			Diabetic retinopathy, blindness			Residual causes of vision loss, MSVI			Residual causes of vision loss, blindness		
Central Europe, Eastern Europe, and Central Asia	0·252 (0·178–0·341)	0·432 (0·307–0·573)	0·346 (0·245–0·464)	0·00881 (0·00549–0·0135)	0·0549 (0·0377–0·0788)	0·0329 (0·0226–0·0472)	7·77 (6·67–9·14)	13·8 (11·9–16·2)	10·9 (9·41–12·8)	1·72 (1·48–1·97)	2·29 (1·97–2·61)	2·02 (1·74–2·29)
High-income	0·315 (0·234–0·420)	0·455 (0·332–0·608)	0·386 (0·284–0·513)	0·0830 (0·0572–0·120)	0·212 (0·153–0·293)	0·148 (0·107–0·209)	3·17 (2·73–3·65)	4·19 (3·60–4·87)	3·69 (3·18–4·26)	0·806 (0·697–0·922)	0·809 (0·699–0·927)	0·808 (0·698–0·923)
Latin America and Caribbean	0·712 (0·526–0·956)	0·787 (0·581–1·04)	0·750 (0·555–0·996)	0·429 (0·310–0·592)	0·416 (0·308–0·567)	0·423 (0·309–0·578)	5·88 (5·12–6·78)	6·90 (5·99–7·94)	6·40 (5·55–7·36)	2·72 (2·32–3·10)	2·85 (2·47–3·24)	2·79 (2·40–3·17)
North Africa and Middle East	0·641 (0·471–0·847)	0·829 (0·611–1·09)	0·732 (0·537–0·965)	0·124 (0·0844–0·180)	0·108 (0·0738–0·159)	0·116 (0·0796–0·168)	3·56 (3·07–4·09)	3·81 (3·31–4·38)	3·68 (3·19–4·23)	2·06 (1·77–2·39)	2·14 (1·83–2·48)	2·10 (1·80–2·44)
Southeast Asia, East Asia, and Oceania	0·477 (0·344–0·642)	0·610 (0·444–0·810)	0·543 (0·394–0·727)	0·134 (0·0914–0·195)	0·163 (0·111–0·241)	0·148 (0·101–0·218)	3·93 (3·45–4·47)	5·18 (4·54–5·89)	4·55 (3·98–5·19)	2·54 (2·20–2·87)	2·75 (2·39–3·10)	2·64 (2·29–2·98)
South Asia	0·207 (0·150–0·278)	0·277 (0·201–0·373)	0·241 (0·176–0·325)	0·0871 (0·0598–0·125)	0·127 (0·0884–0·186)	0·107 (0·0737–0·155)	5·54 (4·85–6·32)	6·41 (5·60–7·31)	5·96 (5·24–6·80)	1·65 (1·41–1·90)	1·60 (1·38–1·85)	1·63 (1·4–1·87)
Sub-Saharan Africa	0·128 (0·0937–0·171)	0·167 (0·122–0·221)	0·148 (0·108–0·195)	0·0350 (0·0240–0·0498)	0·0539 (0·0382–0·0774)	0·0446 (0·0317–0·0640)	4·14 (3·63–4·71)	4·40 (3·84–4·98)	4·27 (3·74–4·84)	2·23 (1·95–2·54)	2·31 (2·02–2·61)	2·27 (1·98–2·57)
Global	0·362 (0·267–0·482)	0·469 (0·345–0·620)	0·416 (0·305–0·551)	0·117 (0·0823–0·165)	0·156 (0·111–0·219)	0·136 (0·0968–0·192)	4·55 (3·99–5·16)	5·72 (5·02–6·51)	5·13 (4·50–5·83)	1·98 (1·73–2·24)	2·08 (1·81–2·35)	2·03 (1·77–2·29)

Table 3. Change in all-age, crude prevalence in 2010-2019 to assess the success of the World Health Assembly's global action plan to reduce avoidable vision impairment.

	MSVI: % change between 2010 and 2019			Blindness: % change between 2010 and 2019		
**Causes of vision loss**	**Crude prevalence**	**Number of cases**	**Age-standardized prevalence**	**Crude prevalence**	**Number of cases**	**Age-standardized prevalence**
All cause	10·9 (9·5–12·5)	22·9 (21·2–24·6)	0·2 (-0·7–1·0)	2·2 (1·0–3·3)	13·2 (11·9–14·4)	-9·9 (-10·6—9·2)
GAP "Avoidable" (cataract + URE)	10·2 (8·6–11·9)	22·0 (20·2–23·9)	-0·2 (-1·0–0·7)	-2·2 (-3·8—0·7)	8·3 (6·5–10·0)	-14·8 (-16·0—13·8)
Cataract	19·3 (17·6–20·8)	32·1 (30·2–33·8)	1·2 (-0·1–2·4)	-1·5 (-3·3–0·1)	9·1 (7·1–10·8)	-15·2 (-16·5—14·1)
Undercorrected refractive error	5·9 (4·3–7·7)	17·3 (15·5–19·3)	-0·9 (-1·7—0·0)	-5·3 (-6·9—3·8)	4·8 (3·1–6·6)	-13·3 (-14·7—11·8)
Glaucoma	29·4 (27·6–31·3)	43·3 (41·3–45·4)	8·0 (6·5–9·5)	7·2 (5·5–8·7)	18·7 (16·8–20·4)	-10·8 (-11·9—9·9)
Age-related macular degeneration	25·2 (22·9–27·4)	38·6 (36·1–41·1)	5·0 (3·2–6·7)	6·2 (3·6–8·6)	17·6 (14·7–20·3)	-11·7 (-13·8—9·9)
Diabetic retinopathy	15·5 (13·4–17·6)	27·9 (25·6–30·3)	-0·6 (-2·1–0·9)	5·0 (1·6–8·4)	16·2 (12·5–20·0)	-7·6 (-9·8—5·2)
Residual causes of vision loss	11·7 (10·2–13·4)	23·7 (22·0–25·5)	0·7 (-0·1–1·6)	6·6 (5·0–8·3)	18·0 (16·3–19·9)	-2·4 (-3·5—1·2)

Figure 1. Coverage of data sources (1980-2018) with cause-specific vision impairment prevalence data for cataract, age-related macular degeneration, glaucoma and diabetic retinopathy (coverage is presented as numbers of sources per region).

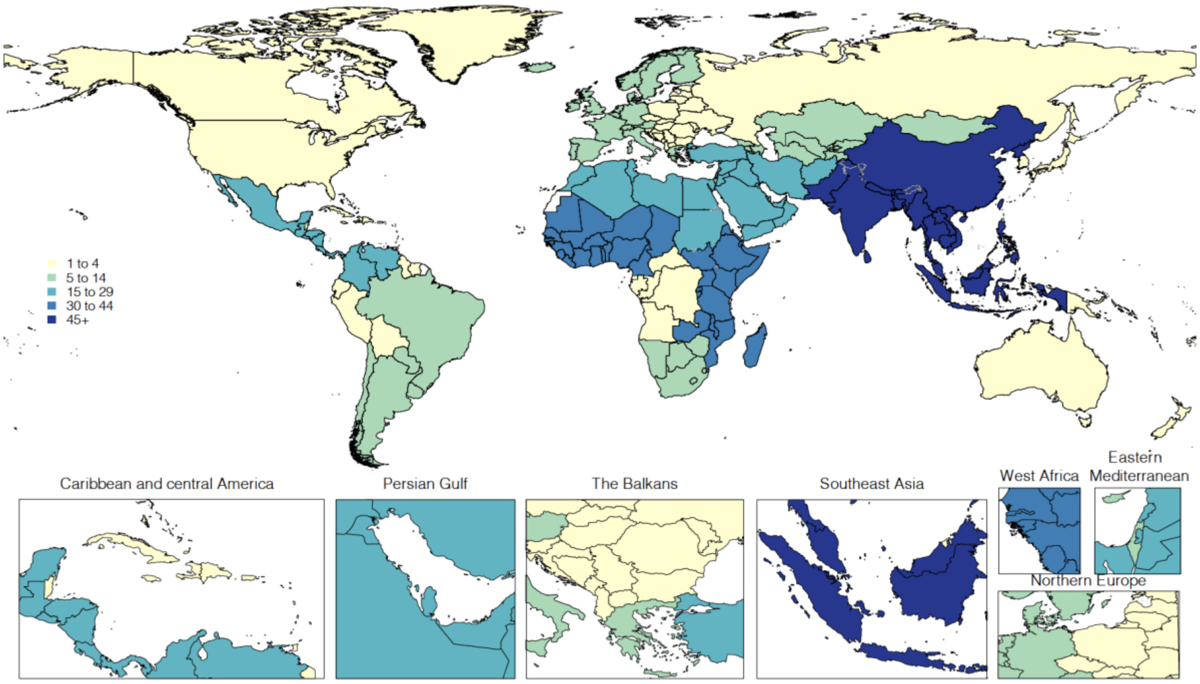

Figure 2. Change in regional age-standardized prevalence of moderate and severe vision impairment between 1990 and 2020 by cause

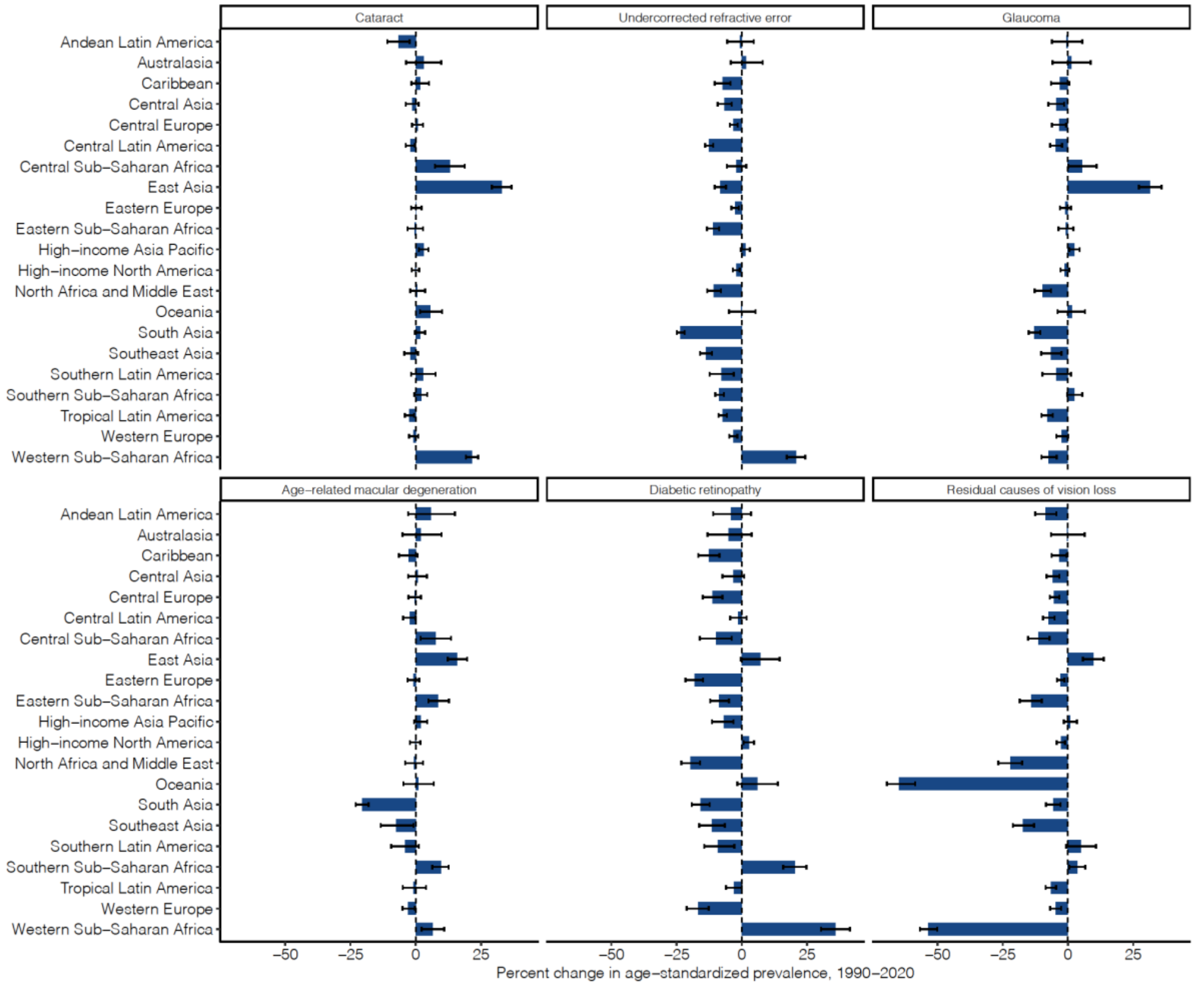

GBD 2019 Blindness Causes Collaborator Contributions

Managing the estimation process

Rupert R A Bourne and Theo Vos.

Writing the first draft of the manuscript

Rupert R A Bourne, Hugh R Taylor, Serge Resnikoff, Robert J Casson, and Jost B Jonas.

Providing data or critical feedback on data sources

Jaimie D Steinmetz, Rupert R A Bourne, Paul Svitil Briant, Seth Flaxman, Hugh R Taylor, Jost B Jonas, Amir Abdoli, Tadele Girum Adal, Elham Ashrafi, Eleni Bagli, Till Winfried Bärnighausen, Akshaya Srikanth Bhagavathula, Mukharram Bikbov, Tasanee Braithwaite, Alain M Bron, Sharath Burugina Nagaraja, Robert James Casson, Ching-Yu Cheng, Dinh-Toi Chu, Maria Vittoria Cicinelli, João M Coelho, Lalit Dandona, Rakhi Dandona, Nikolaos Dervenis, Jenny P Deva, Joshua R Ehrlich, Rajesh Elayedath, Leon B Ellwein, Mohammad Hassan Emamian, Farshad Farzadfar, João M Furtado, Shilpa Gaidhane, Gus Gazzard, Ronnie George, Ahmad Ghashghaee, Mahaveer Golechha, Abdiwahab Hashi, Hung Chak Ho, Mowafa Househ, Seyed Sina Naghibi Irvani, Sheikh Mohammed Shariful Islam, Shubha Jayaram, Rim Kahloun, Taras Kavetskyy, Gbenga A Kayode, John H Kempen, Rovshan Khalilov, Ejaz Ahmad Khan, Rohit C Khanna, Mahalaqua Nazli Khatib, Tawfik Ahmed Muthafer Khoja, Gyu Ri Kim, Judy E Kim, Yun Jin Kim, Sezer Kisa, Adnan Kisa, Soewarta Kosen, Burcu Kucuk Bicer, Van Charles Lansingh, Janet L Leasher, Nicolas Leveziel, Hans Limburg, Xuefeng Liu, Shilpashree Madhava Kunjathur, Navid Manafi, Kaweh Mansouri, Colm McAlinden, Ted R Miller, Seyed Farzad Mohammadi, Jemal Abdu Mohammed, Ali H Mokdad, Getahun Fentaw Mulaw, Kovin S Naidoo, Vinay Nangia, Sreenivas Narasimha Swamy, Huong Lan Thi Nguyen, Felix Akpojene Ogbo, Andrew T Olagunju, Obinna E Onwujekwe, Mayowa O Owolabi, Keyvan Pakshir, Songhomitra Panda-Jonas, Utsav Parekh, Eun-Cheol Park, Shrikant Pawar, Konrad Pesudovs, Tunde Peto, Hai Quang Pham, Vivek Podder, Vafa Rahimi-Movaghar, David Laith Rawaf, Salman Rawaf, Lal Rawal, Nickolas Reinig, Andre M N Renzaho, Alan L Robin, Luca Rossetti, Siamak Sabour, Sare Safi, Abdallah M Samy, Brijesh Sathian, Mete Saylan, Amira A Shaheen, Masood Ali Shaikh, Tueng T Shen, Jae Il Shin, Juan Carlos Silva, Jasvinder A Singh, Deepika Singhal, Eirini Skiadaresi, Valentin Yurievich Skryabin, Anna Aleksandrovna Skryabina, Amin Soheili, Muluken Bekele Sorrie, Chandrashekhar T Sreeramareddy, Nina Tahhan, Bach Xuan Tran, Gebiyaw Wudie Tsegaye, Avina Vongpradith, Giang Thu Vu, Ya Xing Wang, Ningli Wang, Sheila K West West, Temesgen Gebeyehu Wondmeneh, Mehdi Yaseri, Naohiro Yonemoto, Chuanhua Yu, Mikhail Sergeevich Zastrozhin, Anasthasia Zastrozhina, Zhi-Jiang Zhang, Stephanie R M Zimsen, and Theo Vos.

Development of methods or computational machinery

Jaimie D Steinmetz, Rupert R A Bourne, Hugh R Taylor, Jost B Jonas, Sayyed Amirpooya Alemzadeh, Akshaya Srikanth Bhagavathula, Michele Bottone, Paul Svitil Briant, Elizabeth A Cromwell, Seth Flaxman, Mowafa Househ, Ayele Semachew Kasa, Sezer Kisa, Adnan Kisa, Hans Limburg, Ali H Mokdad, Getahun Fentaw Mulaw, Homa Naderifar, Kovin S Naidoo, Songhomitra Panda-Jonas, Vivek Podder, Alan L Robin, Abdallah M Samy, Temesgen Gebeyehu Wondmeneh, and Theo Vos.

Providing critical feedback on methods or results

Jaimie D Steinmetz, Rupert R A Bourne, Paul Svitil Briant, Seth Flaxman, Hugh R Taylor, Jost B Jonas, Woldu Aberhe Abrha, Ahmed Abualhasan, Eman Abu-Gharbieh, Tadele Girum Adal, Hamid Ahmadieh, Wondu Alemayehu, Sayyed Amirpooya Alemzadeh, Ahmed Samir

Alfaar, Vahid Alipour, Jalal Arabloo, Brhane Berhe Aregawi, Alessandro Arrigo, Elham Ashrafi, Desta Debalkie Atnafu, Eleni Bagli, Till Winfried Bärnighausen, Akshaya Srikanth Bhagavathula, Nikha Bhardwaj, Pankaj Bhardwaj, Krittika Bhattacharyya, Ali Bijani, Michele Bottone, Tasanee Braithwaite, Alain M Bron, Sharath Burugina Nagaraja, Zahid A Butt, Florentino Luciano Caetano dos Santos, Vera L A Carneiro, Robert James Casson, Jee-Young Jasmine Choi, Dinh-Toi Chu, João M Coelho, Nathan G Congdon, Rosa A S Couto, Elizabeth A Cromwell, Saad M A Dahlawi, Xiaochen Dai, Lalit Dandona, Rakhi Dandona, Monte A Del Monte, Meseret Derbew Molla, Nikolaos Dervenis, Abebaw Alemayehu Desta, Daniel Diaz, Shirin Djalalinia, Joshua R Ehrlich, Rajesh Elayedath, Hala Rashad Elhabashy, Mohammad Hassan Emamian, Sharareh Eskandarieh, Farshad Farzadfar, Florian Fischer, João M Furtado, Shilpa Gaidhane, Berhe Gebremichael, Ahmad Ghashghaee, Syed Amir Gilani, Mahaveer Golechha, Risky Kusuma Hartono, Abdiwahab Hashi, Simon I Hay, Khezar Hayat, Golnaz Heidari, Hung Chak Ho, Ramesh Holla, Mowafa Househ, John J Huang, Segun Emmanuel Ibitoye, Irena M Ilic, Milena D Ilic, Seyed Sina Naghibi Irvani, Sheikh Mohammed Shariful Islam, Ramaiah Itumalla, Shubha Jayaram, Ravi Prakash Jha, Rohollah Kalhor, Himal Kandel, Ayele Semachew Kasa, Taras Kavetskyy, Gbenga A Kayode, John H Kempen, Rovshan Khalilov, Ejaz Ahmad Khan, Rohit C Khanna, Mahalaqua Nazli Khatib, Tawfik Ahmed Muthafer Khoja, Gyu Ri Kim, Yun Jin Kim, Sezer Kisa, Adnan Kisa, Ai Koyanagi, Burcu Kucuk Bicer, Vaman Kulkarni, Om P Kurmi, Van Charles Lansingh, Janet L Leasher, Xuefeng Liu, Shilpashree Madhava Kunjathur, Navid Manafi, Colm McAlinden, Gebrekiros Gebremichael Meles, Abera M Mersha, Irmina Maria Michalek, Ted R Miller, Sanjeev Misra, Yousef Mohammad, Seyed Farzad Mohammadi, Jemal Abdu Mohammed, Ali H Mokdad, Mohammad Ali Moni, Getahun Fentaw Mulaw, Mehdi Naderi, Kovin S Naidoo, Mukhammad David Naimzada, Vinay Nangia, Sreenivas Narasimha Swamy, Muhammad Naveed, Hadush Negash, Huong Lan Thi Nguyen, Felix Akpojene Ogbo, Andrew T Olagunju, Obinna E Onwujekwe, Nikita Otstavnov, Mayowa O Owolabi, Songhomitra Panda-Jonas, Utsav Parekh, Eun-Cheol Park, Maja Pasovic, Shrikant Pawar, Konrad Pesudovs, Tunde Peto, Hai Quang Pham, Marina Pinheiro, Vivek Podder, Vafa Rahimi-Movaghar, Mohammad Hifz Ur Rahman, Priya Rathi, David Laith Rawaf, Salman Rawaf, Lal Rawal, Andre M N Renzaho, Aziz Rezapour, Alan L Robin, Siamak Sabour, Sare Safi, Mohammad Ali Sahraian, Abdallah M Samy, Brijesh Sathian, Ganesh Kumar Saya, Mete Saylan, Masood Ali Shaikh, Wondimeneh Shibabaw Shiferaw, Mika Shigematsu, Jae Il Shin, Jasvinder A Singh, Deepika Singhal, Rita S Sitorus, Eirini Skiadaresi, Valentin Yurievich Skryabin, Anna Aleksandrovna Skryabina, Amin Soheili, Muluken Bekele Sorrie, Raúl A R C Sousa, Chandrashekhar T Sreeramareddy, Eyayou Girma Tadesse, Md Ismail Tareque, Fotis Topouzis, Bach Xuan Tran, Gebiyaw Wudie Tsegaye, Giang Thu Vu, Ningli Wang, Abrha Hailay Weldemariam, Sheila K West West, Temesgen Gebeyehu Wondmeneh, Mehdi Yaseri, Naohiro Yonemoto, Chuanhua Yu, Mikhail Sergeevich Zastrozhin, Anasthasia Zastrozhina, Zhi-Jiang Zhang, Serge Resnikoff, and Theo Vos.

Extracting, cleaning, or cataloging data; designing or coding figures and tables

Jaimie D Steinmetz, Rupert R A Bourne, Paul S Briant, Seth Flaxman, Jost B Jonas, Mukharram Bikbov, Michele Bottone, Tasanee Braithwaite, Alain M Bron, Ching-Yu Cheng, Maria Vittoria Cicinelli, Arthur G Fernandes, João M Furtado, Ronnie George, Rim Kahloun, John H Kempen, Moncef Khairallah, Van Charles Lansing, Janet L Leasher, Hans Limburg, Vinay Nangia, Kolawole Ogundimo, Konrad Pesudovs, Tunde Peto, Nickolas Reinig, Alexander Silvester, Nina Tahhan, Ningli Wang, Tien Y Wong, Serge Resnikoff, and Theo Vos.

Drafting the manuscript or revising it critically for important intellectual content

Jaimie D Steinmetz, Rupert R A Bourne, Paul Svitil Briant, Seth Flaxman, Hugh R Taylor, Jost B Jonas, Ahmed Abualhasan, Eman Abu-Gharbieh, Tadele Girum Adal, Wondu Alemayehu, Sayyed Amirpooya Alemzadeh, Ahmed Samir Alfaar, Sofia Androudi, Jalal Arabloo, Brhane Berhe Aregawi, Alessandro Arrigo, Eleni Bagli, Atif Amin Baig, Till Winfried Bärnighausen, Maurizio Battaglia Parodi, Mahya Beheshti, Krittika Bhattacharyya, Michele Bottone, Tasanee Braithwaite, Alain M Bron, Sharath Burugina Nagaraja, Vera L A Carneiro, Robert James Casson, Jee-Young Jasmine Choi, João M Coelho, Nathan G Congdon, Reza Dana, Monte A Del Monte, Meseret Derbew Molla, Nikolaos Dervenis, Abebaw Alemayehu Desta, Daniel Diaz, Joshua R Ehrlich, Mohammad Hassan Emamian, Sharareh Eskandarieh, Florian Fischer, David S Friedman, João M Furtado, Shilpa Gaidhane, Gus Gazzard, Berhe Gebremichael, Ahmad Ghashghaee, Samer Hamidi, Billy Randall Hammond, Abdiwahab Hashi, Golnaz Heidari, Ramesh Holla, Mowafa Househ, Segun Emmanuel Ibitoye, Irena M Ilic, Milena D Ilic, Seyed Sina Naghibi Irvani, Sheikh Mohammed Shariful Islam, Ramaiah Itumalla, Shubha Jayaram, Ravi Prakash Jha, Taras Kavetskyy, Gbenga A Kayode, John H Kempen, Rovshan Khalilov, Ejaz Ahmad Khan, Rohit C Khanna, Mahalaqua Nazli Khatib, Tawfik Ahmed Muthafer Khoja, Gyu Ri Kim, Sezer Kisa, Ai Koyanagi, Vaman Kulkarni, Om P Kurmi, Iván Landires, Van Charles Lansingh, Janet L Leasher, Kate E LeGrand, Shilpashree Madhava Kunjathur, Shokofeh Maleki, Navid Manafi, Colm McAlinden, Yousef Mohammad, Jemal Abdu Mohammed, Ali H Mokdad, Mohammad Ali Moni, Ahmed Al Montasir, Mehdi Naderi, Kovin S Naidoo, Mukhammad David Naimzada, Vinay Nangia, Sreenivas Narasimha Swamy, Hadush Negash, Huong Lan Thi Nguyen, Virginia Nunez-Samudio, Felix Akpojene Ogbo, Kolawole Ogundimu, Andrew T Olagunju, Obinna E Onwujekwe, Nikita Otstavnov, Mayowa O Owolabi, Songhomitra Panda-Jonas, Eun-Cheol Park, Maja Pasovic, Shrikant Pawar, Konrad Pesudovs, Tunde Peto, Hai Quang Pham, Vivek Podder, Vafa Rahimi-Movaghar, Mohammad Hifz Ur Rahman, Priya Rathi, David Laith Rawaf, Salman Rawaf, Lal Rawal, Siamak Sabour, Amirhossein Sahebkar, Mohammad Ali Sahraian, Abdallah M Samy, Ganesh Kumar Saya, Mete Saylan, Masood Ali Shaikh, Kenji Shibuya, Mika Shigematsu, Jae Il Shin, Jasvinder A Singh, Deepika Singhal, Eirini Skiadaresi, Valentin Yurievich Skryabin, Anna Aleksandrovna Skryabina, Muluken Bekele Sorrie, Raúl A R C Sousa, Chandrashekhar T Sreeramareddy, Dwight Stambolian, Eyayou Girma Tadesse, Bach Xuan Tran, Miltiadis K Tsilimbaris, Rohit Varma, Gianni Virgili, Giang Thu Vu, Ningli Wang, Naohiro Yonemoto, Zhi-Jiang Zhang, Serge Resnikoff, and Theo Vos.

Management of the overall research enterprise

Jaimie D Steinmetz, Rupert R A Bourne, Paul Svitil Briant, Seth Flaxman, Ashkan Afshin, Charlie Ashbaugh, Lalit Dandona, Ali H Mokdad, Hugh R Taylor, Serge Resnikoff, and Theo Vos.
